# Transcriptome dynamics in *Artemisia annua* provides new insights into cold adaptation and de-adaptation

**DOI:** 10.3389/fpls.2024.1412416

**Published:** 2024-08-29

**Authors:** Yunxiao He, Yujiao Zhang, Jiangnan Li, Zhiyi Ren, Wenjing Zhang, Xianghua Zuo, Wei Zhao, Ming Xing, Jian You, Xia Chen

**Affiliations:** ^1^ National and Local United Engineering Laboratory for Chinese Herbal Medicine Breeding and Cultivation, School of Life Sciences, Jilin University, Changchun, Jilin, China; ^2^ Yanbian Korean Autonomous Prefecture Academy of Agricultural Sciences, Yanbian, Jilin, China

**Keywords:** carbon transport, nitrogen transport, cold adaptation, de-adaptation, transcriptomic

## Abstract

Plants adapt to cold stress through a tightly regulated process involving metabolic reprogramming and tissue remodeling to enhance tolerance within a short timeframe. However, the precise differences and interconnections among various organs during cold adaptation remain poorly understood. This study employed dynamic transcriptomic and metabolite quantitative analyses to investigate cold adaptation and subsequent de-adaptation in *Artemisia annua*, a species known for its robust resistance to abiotic stress. Our findings revealed distinct expression patterns in most differentially expressed genes (DEGs) encoding transcription factors and components of the calcium signal transduction pathway within the two organs under cold stress. Notably, the long-distance transport of carbon sources from source organs (leaves) to sink organs (roots) experienced disruption followed by resumption, while nitrogen transport from roots to leaves, primarily in the form of amino acids, exhibited acceleration. These contrasting transport patterns likely contribute to the observed differences in cold response between the two organs. The transcriptomic analysis further indicated that leaves exhibited increased respiration, accumulated anti-stress compounds, and initiated the ICE-CBF-COR signaling pathway earlier than roots. Differential expression of genes associated with cell wall biosynthesis suggests that leaves may undergo cell wall thickening while roots may experience thinning. Moreover, a marked difference was observed in phenylalanine metabolism between the two organs, with leaves favoring lignin production and roots favoring flavonoid synthesis. Additionally, our findings suggest that the circadian rhythm is crucial in integrating temperature fluctuations with the plant’s internal rhythms during cold stress and subsequent recovery. Collectively, these results shed light on the coordinated response of different plant organs during cold adaptation, highlighting the importance of inter-organ communication for successful stress tolerance.

## Introduction

In recent years, the frequency and severity of atypical cold waves have increased, significantly impacting plant vigor and growth (Baumann, 2017; Guo et al., 2018). Consequently, the study of cold tolerance in plants has become a prominent research area within botany. Perennial plants have evolved cold acclimation mechanisms through the long-term evolutionary adaptation of their natural growth processes. Conversely, annual plants require rapid enhancements in cold tolerance within a short timeframe to ensure survival (Wingler, 2015; Liu et al., 2020; Baker et al., 2021; Liu et al., 2022). To combat low temperatures and acquire freezing tolerance, plants have developed a mechanism known as cold adaptation. This process involves a substantial reprogramming of cellular metabolism and a remodeling of tissue architecture (Shi et al., 2018). Cold adaptation is characterized by a series of progressive changes, including the accumulation of specific proteins, alterations in membrane properties, the modulation of signal transduction pathways, the regulation of osmotic balance, the remodeling of cell walls, and the induction of endogenous hormones (Janská et al., 2010; Zhu, 2016; Shi et al., 2018; Barajas-Lopez et al., 2021; Kidokoro et al., 2022; Kutsuno et al., 2022). Notably, de-adaptation after adaptation is also essential for plant survival and growth, although this aspect is often overlooked in research.

To elucidate the molecular mechanisms underlying cold stress responses, numerous studies have employed *Arabidopsis thaliana* as a model organism to investigate its molecular repertoire. These studies have identified classical cold response pathways involving C-repeat/DREB binding factors (CBFs) and have provided strong evidence for the critical role of CBFs in diverse plant species and tissues (Lee et al., 2005; Shi et al., 2018). In *Arabidopsis*, AtCBF1-3 transcription factors are estimated to regulate 12%–20% of cold-induced transcripts (Vogel et al., 2004). Research has progressively unveiled the upstream regulatory elements and downstream targets of CBFs (Jiang et al., 2017; Liu and Zhou, 2018). Additionally, abscisic acid (ABA) and jasmonic acid (JA) have been implicated in plant cold tolerance (Kidokoro et al., 2022). The accumulation of both ABA and JA has been demonstrated to positively regulate freezing tolerance through a CBF-dependent pathway (Hu et al., 2013). Notably, temperature is one of the key factors influencing circadian rhythms, and the circadian clock itself acts as a gatekeeper for the cold response (Chang et al., 2020). Genes associated with the circadian clock can modulate the expression of cold-responsive genes, including CBFs and cold-regulated (COR) genes (Mikkelsen and Thomashow, 2009).

While research has illuminated the expression patterns of key genes in response to low temperatures, it is well-recognized that cold stress responses exhibit significant variation across plant species, growth stages, and organs (Zhu et al., 2013; Jaikumar et al., 2016; Willick et al., 2018; Li et al., 2019; Vergara et al., 2021). For instance, a major physiological effect of cold responses in leaves is the reduction of photosynthesis (Burnett et al., 2022). Consequently, the translocation and metabolic processes of carbohydrates, the direct byproducts of photosynthesis, become tightly regulated within various organs upon exposure to low temperatures (Lemoine et al., 2013; Sperling et al., 2017; Patzke et al., 2019). Roots are also sensitive to cold stress, experiencing growth restrictions that limit water and nutrient uptake, particularly nitrogen. This limitation in nitrogen acquisition can have downstream consequences, as nitrogen availability is essential for various physiological and biochemical processes in plants (Soualiou et al., 2023). Notably, nitrogen is a macronutrient crucial for plant growth and serves as the primary building block for proteins, amino acids, and hormones (Kong et al., 2017; Peng et al., 2021a). Additionally, it plays a critical role in chlorophyll content, photosynthetic capacity, and carbohydrate accumulation (Wang et al., 2021; Yue et al., 2021). Certain carbohydrates and amino acids, such as raffinose, trehalose, fructose, proline, and glycine, are recognized as osmoprotectants. These osmoprotectants, widely distributed in plant species under cold stress, are believed to play a vital role in stabilizing proteins and cellular structures (Sami et al., 2016; Zhang et al., 2022). However, the specific differences and interactions between leaves and roots, as well as their potential synergistic effects under cold conditions, remain poorly understood.

*Artemisia annua*, a member of the Asteraceae family, is renowned for its medicinal value, particularly in treating malaria (Weathers et al., 2010). This annual plant thrives in temperate climates with relative ease of cultivation and exhibits remarkable resistance to both drought and cold stress (Vashisth et al., 2018). However, due to geographical constraints, *A. annua* seedlings in northern China frequently encounter low-temperature environments during the early and late spring seasons. Despite its demonstrated cold tolerance, the underlying mechanisms enabling this robust adaptation in *A. annua* remain largely uncharacterized.

To address these knowledge gaps, we employed a comparative approach utilizing physiological, transcriptomic, and metabolite quantitative analyses of *A. annua* leaves and roots exposed to cold stress and subsequent recovery treatment. Our findings contribute significantly to a deeper understanding of plant adaptation mechanisms during cold stress and provide valuable scientific knowledge that can inform future efforts to enhance cold tolerance in plants.

## Materials and methods

### Plant material and treatment conditions

*A. annua* cultivar Huhao, a high artemisinin-producing variety originating from YouYang, was selected for this study. *A. annua* seeds were procured from the Institute of Botany, Chinese Academy of Sciences. Throughout the experiment, control plants were maintained under a constant temperature of 24°C day/22°C night. Plants designated for cold treatment were transferred to a pre-cooled growth chamber set at 4°C day/4°C night. Seedlings of *A. annua* grown under control conditions for 32 days, 33 days, 34 days, 35 days, 42 days, and 49 days were designated as ND32, ND33, ND34, ND35, ND42, and ND49, respectively. The designations CH6, CD2, CD5, and CD7 represent 35-day-old *A. annua* seedlings subjected to continuous cold treatment for 6 hours, 2 days, 5 days, and 7 days, respectively. The control group (35-day-old *A. annua* seedlings grown for 6 hours under control conditions) was designated as NH6. Seedlings designated as RH6, RD2, and RD7 represent *A. annua* seedlings returned to control conditions for 6 hours, 2 days, and 7 days following 7 days of cold treatment, respectively. Three biological replicates were generated for both leaf and root transcriptome samples, while six replicates were generated for biochemical analysis. At each sampling point, a minimum of six individual plants were collected. All samples were rapidly frozen in liquid nitrogen upon collection and stored at −80°C for subsequent RNA sequencing and biochemical analyses.

### Morphological traits, relative water content, and relative electrolyte conductivity measurements

Following exposure to single or combined stress treatments, 1.5-cm leaf segments were excised from fully expanded leaves. Fresh weight (FW) was immediately measured for each segment. Subsequently, the leaf segments were floated on deionized water within a Petri dish and incubated in darkness at 4°C for 24 hours. Turgid weight (TW) was then determined by re-weighing the segments. Finally, the leaves were oven-dried at 80°C for 48 hours, and dry weight (DW) was measured. Relative water content (RWC) was calculated using the following formula: [(FW − DW)/(TW − DW)] × 100. Relative electrolyte conductivity (REC) was determined using a DDS-307 instrument.

Phenotypic data were acquired from 10 individual plants. Images of the leaves and roots were captured using a Canon EOS R5 camera. Subsequently, leaf area and primary root length were determined using the Adobe Photoshop CC 2018 software.

### Net photosynthetic rate measurement

The net photosynthetic rate (Pn) was measured using a YAXIN-1102 photosynthetic measuring system (Beijing Yaxinliyi Science and Technology) following the manufacturer’s instructions. Prior to measurement, the system was preheated for 30 minutes. Subsequently, it was connected to a transparent tube containing sodium lime for zero-point calibration. To achieve maximum calibration, the system was then connected to a standard CO_2_ gas source with a concentration of 1,000 ppm. A ventilation pipe (6 m in length) was attached to one end of the system, with the other suspended approximately 1.5 m above the ground and positioned away from the researcher. The second fully expanded leaves from the base of the plant were chosen for measurement, with each measurement replicated six times. Environmental parameters during these measurements were maintained consistent with those during sampling. Alternatively, the fifth or sixth fully expanded leaves from the shoot apex could be used for measurement, with each measurement replicated six times.

### Chlorophyll and anthocyanin measurement

Approximately 100 mg of leaf tissue was extracted with 95% ethanol under dark conditions for 24 hours at room temperature. Following extraction, the homogenate was centrifuged at 10,000 × *g* for 10 minutes. The supernatant was then used to measure absorbance at 649 nm and 665 nm using a spectrophotometer. The established formula for calculating the concentrations of chlorophyll *a*, chlorophyll *b*, and total chlorophyll (*a* + *b*) was employed (Sehoefs et al., 2001).

The anthocyanin content was quantified using a modified pH differential spectrophotometric method (Niu et al., 2010). Approximately 300 mg of leaf tissue was pulverized in liquid nitrogen. Subsequently, 1.5 mL of pre-chilled 0.05% HCl in methanol (extraction buffer) was added to the homogenized tissue. Following a 12-hour incubation period in darkness at 4°C, the homogenate was centrifuged at 10,000 × *g* for 20 minutes. The supernatant was collected and transferred to a new 2-mL microtube for storage at 4°C. The extraction process was repeated twice, and the supernatants from both extractions were combined. The absorbance of the combined supernatant (0.3 mL) mixed with 1.2 mL of either buffer A (0.4 M KCl adjusted to pH 1.0 with 2 N HCl) or buffer B (1.2 N citric acid adjusted to pH 4.5 with 0.2 M NaH_2_PO_4_) was measured at 510 nm and 700 nm using a spectrophotometer.

### Antioxidant enzyme assays

Leaf samples (0.1 g) were ground to a fine powder using liquid nitrogen. The resulting powder was then homogenized in 10 mL of extraction buffer. This buffer consisted of 100 mM phosphate buffer (pH 7.0), 1 mM ethylenediaminetetraacetic acid (EDTA), 0.1% Triton-X-100, and 1% polyvinylpyrrolidone (PVP). The homogenate was subsequently centrifuged at 4°C and 12,000 rpm for 20 minutes. The resulting supernatant fraction was collected and used as the enzyme extract for subsequent enzyme assays.

The reaction mixture for superoxide dismutase (SOD) activity comprised 3 mL of 50 mM phosphate buffer (pH 7.3), 13 mM methionine, 75 mM nitroblue tetrazolium, 0.1 mM EDTA, and 4 mM riboflavin. Enzyme extract (0.2 mL) was added to the reaction solution in a glass cuvette and exposed to a 4,000-lux fluorescent lamp for 15 minutes. Blanks and controls were prepared identically but kept in the dark. The absorbance of all samples was measured at 560 nm. One unit of SOD activity was defined as the amount of enzyme required to produce a 50% inhibition of nitroblue tetrazolium reduction under these assay conditions. The peroxidase (POD) reaction mixture contained 50 mM phosphate buffer (pH 7.0), 10 mM hydrogen peroxide (H_2_O_2_), and 10 mM guaiacol. Enzyme extract (20 µL) was added to 180 µL of the reaction solution, and POD activity was measured by monitoring the absorbance change at 470 nm over a 2-minute period. One unit of POD activity was defined as an increase in absorbance of 0.01 per 30 seconds at 470 nm. The catalase (CAT) reaction mixture (180 μL) contained 50 mM phosphate buffer (pH 7.0) and 200 mM H_2_O_2_. The reaction was initiated by adding 20 µL of enzyme extract to a quartz microplate well. Absorbance was recorded at 240 nm every 30 seconds. One unit of CAT activity was defined as an absorbance change of 0.01 per 30 seconds at 240 nm. Ascorbate peroxidase (APX) activity was determined using a modified version of the method described (Dalton et al., 1987). The APX reaction mixture consisted of 50 mM phosphate buffer (pH 7.0), 0.5 mM l-ascorbic acid (LAsA), 0.1 mM EDTA, and 10 mM H_2_O_2_. Enzyme extract (20 µL) was added to 180 µL of the reaction solution, and APX activity was determined by measuring the absorbance decrease at 290 nm for 1 minute. One unit of APX activity was defined as a decrease in absorbance of 0.01 per 30 seconds at 290 nm.

### Quantitative analysis of metabolites

Total organic carbon (TOC) content was quantified using the potassium dichromate oxidation spectrophotometric method (Tang et al., 2020). Total nitrogen (TN) content was determined using a K1300-type Automatic Kjeldahl Apparatus (SONNEN) following the manufacturer’s instructions. Six biological replicates were performed for both TOC and TN analyses.

Free amino acid (FAA) content was determined using the ninhydrin chromogenic method. Free polyamine content was also determined using the ninhydrin chromogenic method. Water-soluble protein (WSP) content was measured using the Biuret method. Lignin content was quantified using the phenolic hydroxyl colorimetric method. Total flavonoid content was determined using the sodium nitrite–aluminum nitrate colorimetric method. Nitrate nitrogen content was measured by the nitrosalicylic acid method, and ammonium nitrogen content was determined by the ninhydrin coloration method. Six biological replicates were performed for these assays using commercially available kits (Solarbio, Beijing, China). Nitrate reductase (NR) and nitrite reductase (NiR) activities were measured using assay kits from Solarbio with six biological replicates per sample.

Water-soluble sugar (WSS) and starch contents were determined following extraction with 1.5 mL of 80% (v/v) ethanol. Samples were incubated at 75°C for 60 minutes, followed by centrifugation at 7,400 × *g* for 10 minutes. The supernatant was collected in a new tube and evaporated to dryness at 45°C. The residue was then reconstituted in 200 µL of water for subsequent WSS determination (six biological replicates). The starch content of the pellet obtained from centrifugation was determined using the same extraction method (six biological replicates). The pellet was washed with ethanol and water before incubation with 0.2 M NaOH at 95°C for 1 hour. Subsequently, 1 M acetic acid was added to adjust the pH to 5.5, followed by starch quantification using the phenol–sulfuric acid method (standard curve: y = 0.0089x + 0.0041, R^2^ = 0.9994). Malondialdehyde (MDA) content was measured using the thiobarbituric acid (TBA) method with six biological replicates (standard curve: y = 59.838x + 1.5288, R^2^ = 0.9942).

### RNA extraction, transcriptome sequencing, library construction, and gene annotation

For RNA sequencing (RNA-seq) analysis, samples were collected from six individual plants and pooled to create a single biological replicate. Three biological replicates were generated in total. Total RNA was extracted from plant leaves using TRIzol reagent (Invitrogen, Burlington, ON, Canada). The integrity and concentration of the isolated RNA were assessed using an Agilent 2100 Bioanalyzer (Agilent Technologies, Inc., Santa Clara, CA, USA). Subsequently, mRNA was isolated using the NEBNext Poly (A) mRNA Magnetic Isolation Module (New England Biolabs Inc., NEB, Ipswich, MA, USA; E7490). The cDNA library construction followed the manufacturer’s instructions for the NEBNext Ultra RNA Library Prep Kit for Illumina (NEB, E4500) and NEBNext Multiplex Oligos for Illumina (NEB, E4500). Briefly, the enriched mRNA was fragmented to generate approximately 200-nucleotide RNA inserts, which served as templates for first- and second-strand cDNA synthesis. The double-stranded cDNA underwent end-repair, dA-tailing, and adaptor ligation. Fragments of appropriate size were purified using Agencourt AMPure XP beads (Beckman Coulter, Inc., Brea, CA, USA) and enriched through PCR amplification. Finally, the constructed cDNA libraries of *A. annua* were sequenced on an Illumina HiSeq™ sequencing platform. Clean reads, obtained after filtering raw reads, were mapped to the *A. annua* genome using the Tophat2 software (Kim et al., 2013; Shen et al., 2018). Aligned reads in BAM/SAM format were further processed to remove potential duplicate sequences.

### Identification and analysis of differentially expressed genes

Clean reads, obtained after filtering raw reads, were mapped to the *A. annua* genome using the Tophat2 software (Trapnell et al., 2010). Differential expression analysis was performed using the DESeq2 software (Love et al., 2014). Q-values were employed to assess the statistical significance of the identified differentially expressed genes (DEGs). Gene abundance differences between samples were then determined based on the fold change (FC) in their normalized expression values [fragments per kilobase million (FPKM)]. To account for multiple testing, the false discovery rate (FDR) control method was utilized to establish a statistically significant threshold for the *p*-value. Consequently, only genes exhibiting an absolute log_2_ fold change (|log2FC|) ≥ 1 and an FDR significance score < 0.05 were retained for further analysis.

Gene sequences were subjected to similarity searches against various protein databases using the BLASTX algorithm. These databases included the National Center for Biotechnology Information (NCBI) non-redundant protein (Nr) database and the Swiss-Prot protein database. A stringent E-value cutoff of 1e^−5^ was employed to filter the BLASTX results. Additionally, BLASTn searches were performed against the NCBI non-redundant nucleotide sequence (Nt) database using the same E-value cutoff (1e^−5^). Genes were assigned putative functions based on the best BLAST hit (highest score) and its associated protein functional annotation. For the Gene Ontology (GO) term assignment, the results from the Nr BLAST searches were imported into the Blast2GO program (Conesa et al., 2005). Blast2GO retrieved GO annotations for the genes by mapping them to corresponding terms within its database. This process allowed categorizing annotated genes based on their biological process, cellular component, and molecular function. Subsequently, a Perl script was utilized to visualize the distribution of gene functions across different GO categories for those unigenes with assigned GO terms. To further refine and enrich the obtained functional annotations, the TopGO (R package) was employed. Furthermore, gene sequences were aligned to the Clusters of Orthologous Groups (COG) database to predict and classify their putative functions (Tatusov et al., 2000). Finally, the Kyoto Encyclopedia of Genes and Genomes (KEGG) pathway assignments for the assembled sequences were performed using a custom Perl script.

### Quantitative real-time polymerase chain reaction

Quantitative real-time PCR (qRT-PCR) was employed to validate the transcriptome results obtained in this study. The first-strand cDNA synthesis was performed using the FastQuant RT Kit (TIANGEN, Beijing, China). RNA concentration and integrity were assessed using a Nanodrop 2100 spectrophotometer and 1.0% agarose gel electrophoresis, respectively. Each qRT-PCR contained approximately 1.5 ng of cDNA template and targeted a specific gene. The Actin gene served as an internal control for normalization, and the ΔΔCt method was used for relative quantification. All qRT-PCRs were performed using the SYBR Green Fast kit (ABclonal Technology, Woburn, MA, USA) on a 7500 Real-Time PCR system (Applied Biosystems, now Thermo Fisher Scientific, Waltham, MA, USA) with three technical replicates. Primers used for qRT-PCR are listed in **Supplementary Table S9**.

### Statistical analysis

Statistical analyses were performed using the SPSS software version 26.0 (SPSS Inc., Chicago, IL, USA). Duncan’s test was employed for *post-hoc* multiple comparison analysis. Differential expression analysis was conducted using the DESeq2 package within the R statistical environment (version 4.2.1). To control for FDR, resulting *p*-values were adjusted using the Benjamini–Hochberg procedure. Co-expression network construction was performed using the WGCNA package in R (version 4.2.1). Genes were selected based on the following criteria: FPKM ≥ 10, minimum module size of 30 genes, and FPKM variation ≥ 0.5.

Data visualization was performed in R version 4.2.1. Box plots, line plots, and heatmaps were generated using the ggplot2 and pheatmap packages. Principal component analysis (PCA) was conducted using the gmodels package, and the resulting components were visualized using the ggpubr package.

## Results

### Morphological traits and physiological indicators of *A. annua*

**Figure 1:**
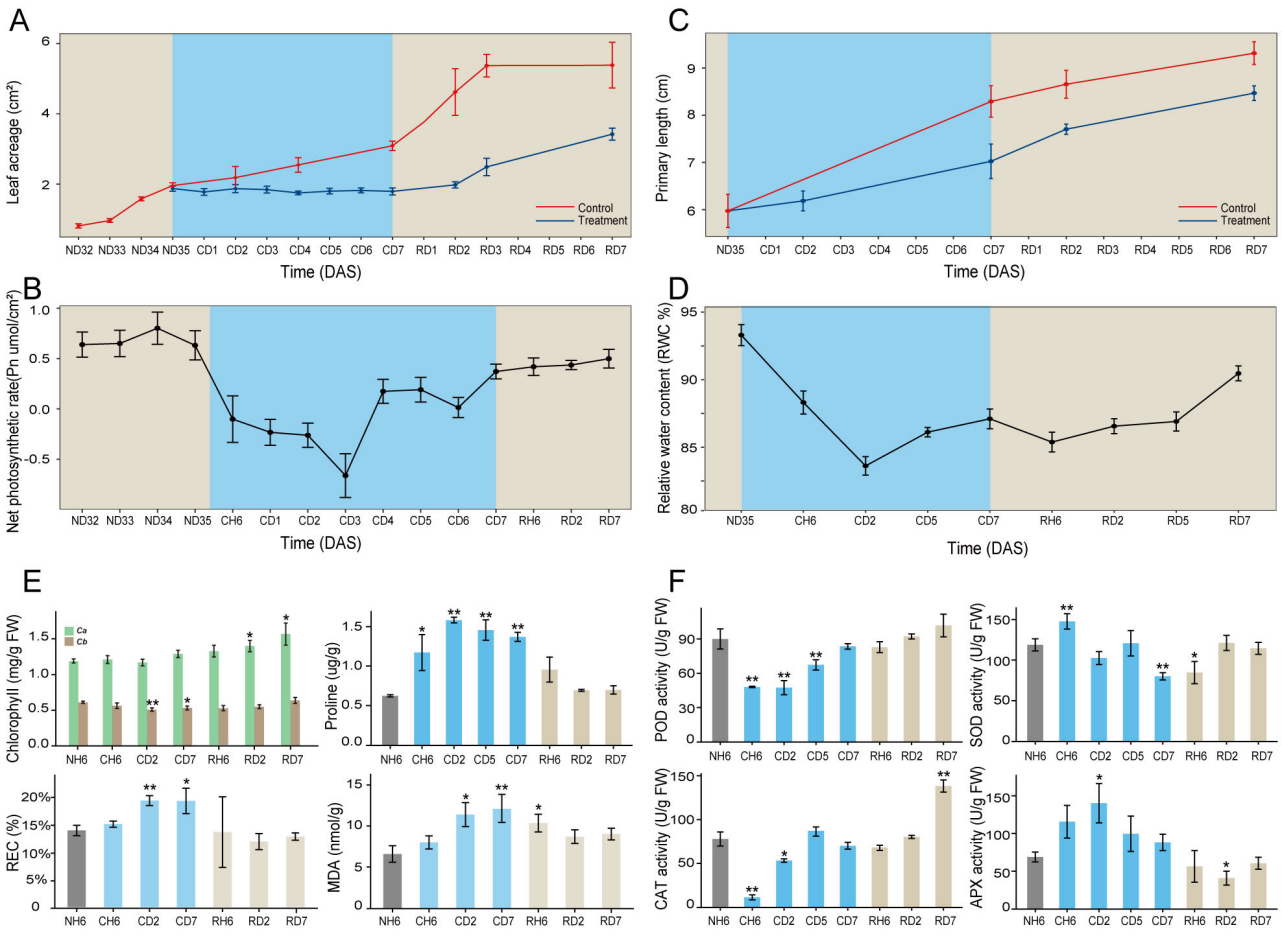
Morphological traits and physiological indicators of *Artemisia annua*. **(A–C)** Dynamic changes of leaf area **(A)**, net photosynthetic rate (Pn; **B**), and primary root length **(C)** throughout the entire treatment. **(D)** Dynamics of relative water content (RWC%) in leaves. **(E)** Chlorophyll, proline, relative electrolyte leakage rate (REC), and malondialdehyde (MDA) content of leaves. **(F)** Superoxide dismutase (SOD), peroxidase (POD), catalase (CAT), and ascorbate peroxidase (APX) activities of leaves. The data of leaf area and primary root length are presented as means ± SD (n = 10). Other data are expressed as means ± SD (n = 6). Asterisks indicate significant differences between treatment groups and the control group (NH6). Significance levels are as follows: *p < 0.05; **p < 0.01.

To investigate the cold stress responses of *A. annua*, this study employed a progressive cold treatment approach using this plant species. Initial observations revealed a significant reduction in plant growth following exposure to cold stress conditions (**Figure 1A**). Leaf curling and wilting symptoms were evident at CH6 and CD2 treatment durations but were absent at CD7 (**Supplementary Figure S1A**). The Pn and water content of leaves exhibited an initial decrease followed by a subsequent increase throughout the cold treatment period (**Figures 1B, D**). We further evaluated various physiological indices in *A. annua* following cold stress. Chlorophyll *a* content in leaves displayed no significant changes under cold treatment, as shown in **Figure 1E**. In contrast, chlorophyll *b* content exhibited a significant decrease at CD2 (*p* < 0.01) and CD7 (*p* < 0.05) compared to the control group. Proline content displayed an upward trend relative to the control (NH6). Conversely, both the REC and MDA content increased significantly at CD2 and CD7. Enzyme activity assays (**Figure 1F**) revealed similar trends for POD and CAT activities, with both enzymes showing a significant decrease at CH6 (*p* < 0.01) followed by a recovery at CD5 and CD7. SOD activity displayed an increase at CH6 and a decrease at CD7 (*p* < 0.01). APX activity, however, exhibited an upward trend compared to the control, with a statistically significant increase observed at CD2 (*p* < 0.05). Regarding root development under cold stress conditions, the primary root maintained its growth (**Figure 1C**). However, the development of lateral roots was significantly inhibited, as shown in **Supplementary Figure S1B**.

The above indicators exhibited noteworthy changes, with significant differences observed at CH6, CD2, and CD7 time points (**Figure 1**). These findings highlight the critical importance of these specific time intervals in understanding the cold stress response of *A. annua*. Furthermore, significant variations in the growth rates of both leaves and roots were evident at the RD2 time point. Consequently, additional time points, namely, RH6, RD2, and RD7, were identified as crucial for further investigation within this study design.

### RNA sequencing and quantitative real-time polymerase chain reaction validation

**Figure 2:**
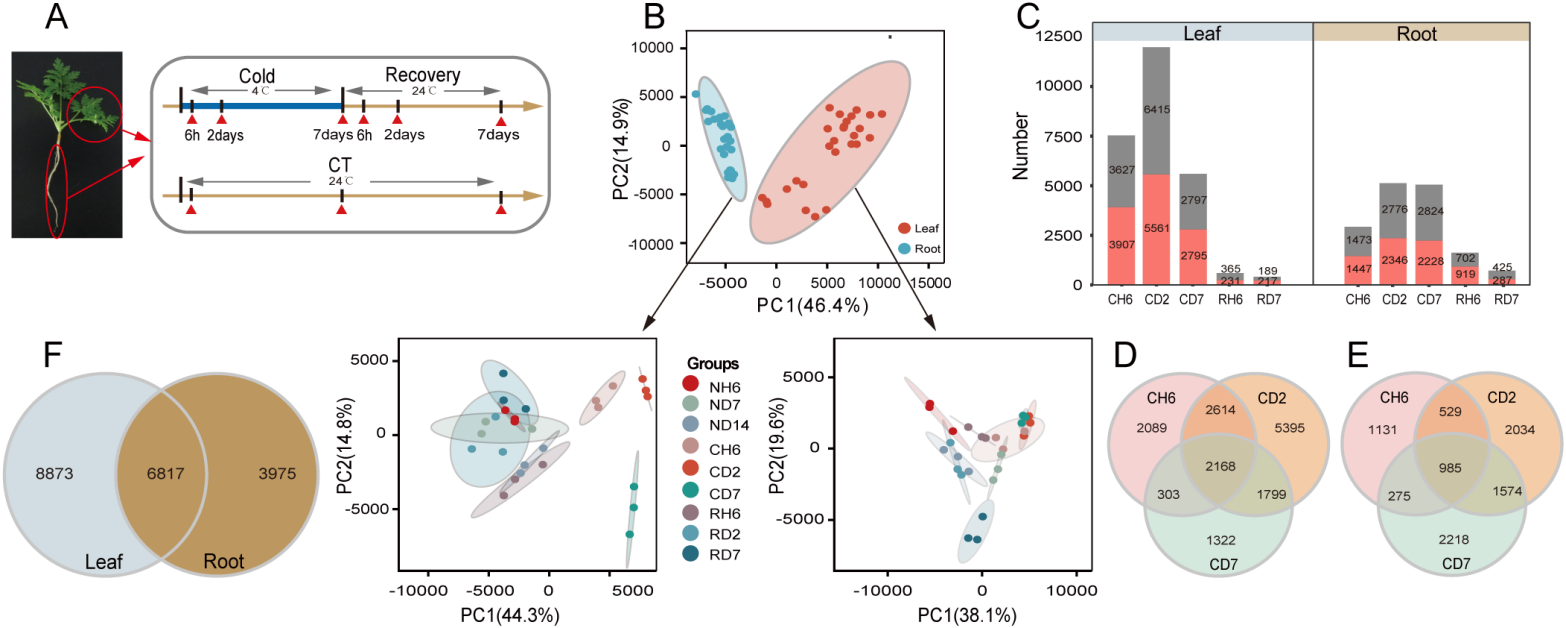
Transcriptome analysis of *Artemisia annua* under cold and recovery treatment. **(A)** A schematic illustration of *A. annua* sampling time points. All samples were used for RNA sequencing (RNA-seq), and each treatment has three biological replicates. **(B)** Principal component analysis based on all genes identified by RNA-seq. PC1, principal component 1; PC2, principal component 2. **(C)** Number of differentially expressed genes (DEGs) [log2 |fold change| ≥ 1, false discovery rate (FDR) < 0.05] identified by RNA-seq. **(D)** Venn diagram representing the number of DEGs in leaves after cold treatment. **(E)** Venn diagram representing the number of DEGs in roots after cold treatment. **(F)** Venn diagram analysis of DEGs in leaves and roots after cold treatment.

Based on observed growth rates and physiological changes, a total of 18 samples were collected for RNA-seq analysis, with three biological replicates for each sample (**Figure 2A**). This resulted in the generation of 54 paired-end libraries, yielding a total of 380.28 gigabytes (Gb) of clean data. On average, each library contained approximately 7.04 Gb of clean data, with a minimum Q30 base percentage of 93.54% (indicating high sequencing quality). Alignment of these sequences to the reference genome of *A. annua* revealed alignment efficiencies ranging from 81.82% to 87.77% (**Supplementary Table S1**). To gain a comprehensive understanding of the cold stress and recovery processes, PCA was performed using all genes identified by RNA-seq. The resulting PCA plot (**Figure 2B**) demonstrated a clear separation between leaf and root samples, further confirming the consistency of biological replicates within each sample group. DEG analysis identified 7,534, 11,976, and 5,592 DEGs in response to CH6, CD2, and CD7 treatments in leaves, respectively (fold change > 2; FDR < 0.05; **Figure 2C**). Similarly, 2,920, 5,122, and 5,052 DEGs were identified in roots under the same cold stress conditions. Notably, DEGs were also identified during subsequent recovery stages (**Supplementary Table S2**). An interesting finding was the presence of a subset of DEGs shared across CH6, CD2, and CD7 treatments. In leaves, 2,168 genes were co-regulated under these conditions, while 985 genes displayed co-regulation in roots (**Figures 2D, E**). Furthermore, a significant proportion of genes (44% in leaves and 63% in roots) exhibited differential expression in both tissues, suggesting potential crosstalk between these organs during the cold stress response (**Figure 2F**).

To validate the RNA-seq data’s accuracy, 10 DEGs were chosen for further analysis using qPCR. These DEGs were selected based on their putative roles in various biological processes, including photoprotection, flavonoid synthesis, unsaturated fatty acid synthesis, circadian rhythms, and alpha-linolenic acid/linoleic acid degradation (**Supplementary Figures S2A, B**). The qPCR results demonstrated a consistent pattern of up- or downregulation for each DEG compared to the RNA-seq data, thereby supporting the validity of the RNA-seq analysis.

### Functional annotation of differentially expressed genes

**Figure 3:**
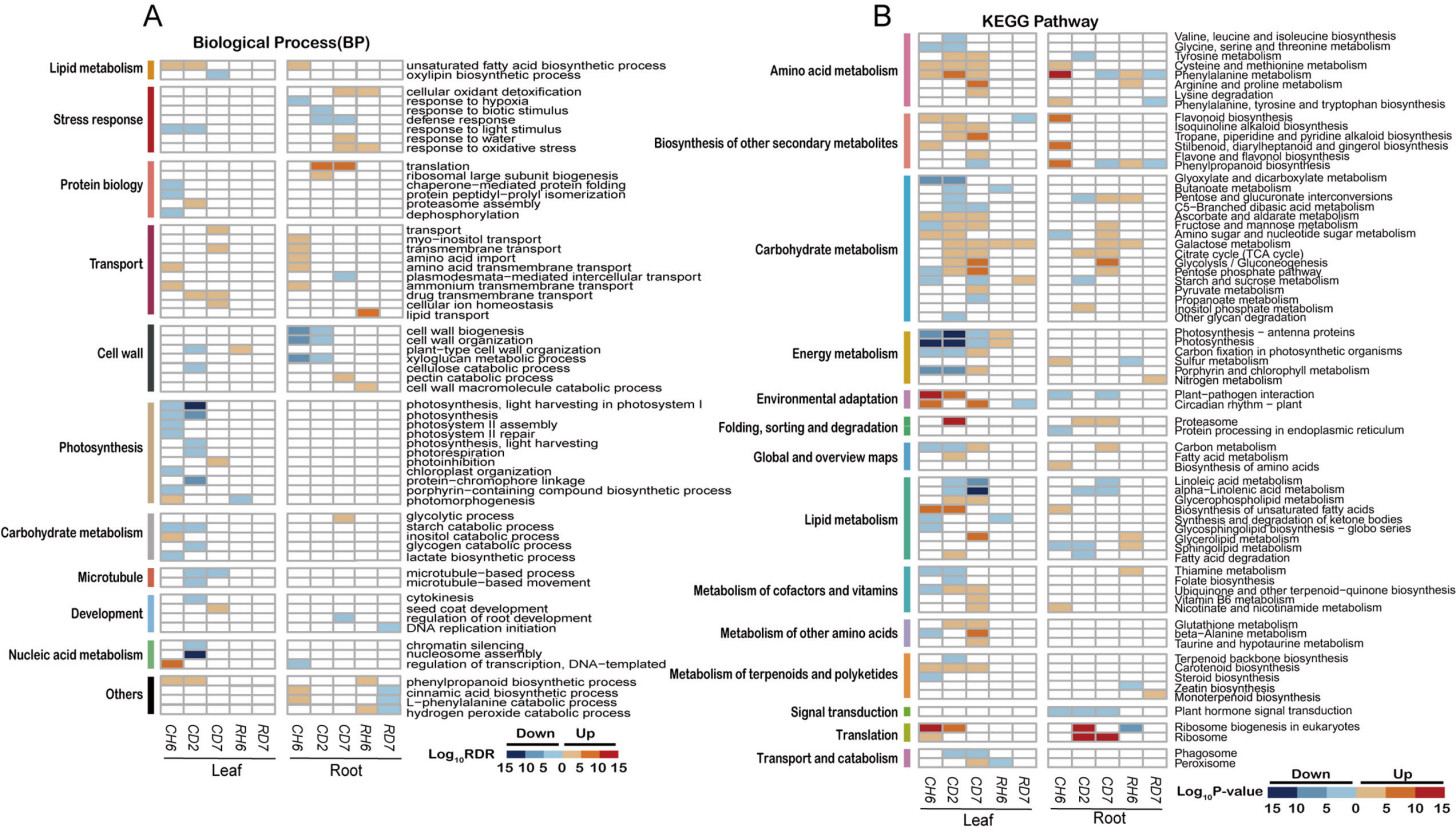
Functional annotations of differentially expressed genes under cold stress. **(A)** Heatmaps illustrating non-redundant Gene Ontology term [GO; biological process; false discovery rate (FDR) < 0.05] enrichment according to DAVID and REVIGO. The color scale corresponds to the −log10 transformation of the FDR for the enrichment according to Fisher’s exact test. **(B)** Heatmaps illustrating Kyoto Encyclopedia of Genes and Genomes (KEGG) pathways (*p* < 0.01) based on DEG enrichment. The color scale corresponds to the −log10 transformation of the *p*-value for the enrichment. The regulatory trend of the transcripts in each bin (up or down) is indicated.

GO enrichment analysis was employed to investigate the potential functional roles of DEGs associated with the cold stress response. A set of 37 consistently enriched terms was identified across the three time points examined in leaves exposed to cold stress. Specific enriched GO categories are presented for each time point (FDR < 0.05; **Figure 3A**). For instance, at CH6, the most significantly enriched category among upregulated genes was “DNA-templated transcription, elongation” (GO:0006354, FDR = 8.01 × 10^−7^). Additional enrichments were observed for “calmodulin binding” (GO:0005516, FDR = 5.25 × 10^−3^) and “protein serine/threonine kinase activity” (GO:0004674, FDR = 3.01 × 10^−2^; **Supplementary Figure S3A**). Downregulated genes at CD2 exhibited particular enrichment for processes related to nucleic acid metabolism, such as “nucleosome assembly” (GO:0006334, FDR = 1.12 × 10^−11^) and “chromatin silencing” (GO:0006342, FDR = 5.35 × 10^−4^). Additionally, enrichments were observed for “microtubule-based movement” (GO:0007018, FDR = 2.10 × 10^−3^) and “cytokinesis” (GO:0000910, FDR = 0.04), suggesting suppression of DNA synthesis and cell proliferation. At CD7, the gene expression landscape shifted, with a majority of upregulated genes clustering within categories related to transport, including “transmembrane transport” (GO:0055085, FDR = 1.23 × 10^−4^) and “drug transmembrane transport” (GO:0006855, FDR = 1.67 × 10^−3^). Notably, GO terms associated with photosynthesis were significantly enriched among the downregulated genes at CH6 and CD2, but not at CD7.

Analysis of roots subjected to cold stress revealed a total of 24 enriched GO terms (FDR < 0.05; **Figure 3A**). At CH6, upregulated genes were primarily associated with transport functions, exhibiting significant enrichment in categories such as “myo-inositol transport” (GO:0015798, FDR = 3.07 × 10^−2^) and “transmembrane transport” (GO:0055085, FDR = 2.70 × 10^−4^). Additional enrichments were observed for “amino acid import” (GO:0043090, FDR = 3.43 × 10^−3^) and “amino acid transmembrane transport” (GO:0003333, FDR = 0.01). Interestingly, pathways related to “unsaturated fatty acid biosynthetic process” (GO:0006636, FDR = 1.02 × 10^−4^) and “cinnamic acid biosynthetic process” (GO:0009800, FDR = 0.02) displayed significant upregulation. Conversely, genes involved in “regulation of transcription, DNA-templated” (GO:0006355, FDR = 0.03) were downregulated. Cell wall-related processes, particularly “cell wall biogenesis” (GO:0042546, FDR = 4.42 × 10^−10^) and “xyloglucan metabolic process” (GO:0010411, FDR = 3.83 × 10^−10^), were enriched among downregulated genes at both CH6 and CD2. At CD2 and CD7, DEGs were significantly enriched in categories associated with translation and stress response. Notably, downregulated genes at CD2 also included those involved in “response to biotic stimulus” (GO:0009607, FDR = 2.43 × 10^−4^) and “defense response” (GO:0006952, FDR = 0.01). Additionally, genes related to “cellular oxidant detoxification” (GO:0098869, FDR = 0.01), “responses to water” (GO:0009415; FDR = 0.01), and “response to oxidative stress” (GO:0006979, FDR = 0.01) were specifically upregulated at CD7.

We further conducted an enrichment analysis of DEGs using the KEGG database (*p* < 0.01; **Figure 3A**). This analysis revealed significant enrichment of DEGs within specific metabolic pathways (*p* < 0.01). Interestingly, certain pathways displayed unique enrichment patterns in leaves compared to roots. Notably, leaves exhibited enrichment in pathways associated with energy metabolism, circadian rhythm, cofactor and vitamin metabolism, amino acid metabolism, and carotenoid biosynthesis. Conversely, roots displayed a higher enrichment in pathways related to carbohydrate metabolism and translation, while also showing enrichment in amino acid metabolism. Additionally, roots displayed enrichment in the biosynthesis of secondary metabolites.

### Analysis of genes encoding Ca^2+^ channel proteins and transcription factors

Transcriptomic data analysis and KEGG pathway enrichment of DEGs revealed many genes putatively involved in signal perception, transduction, and regulation during cold stress. Notably, the “Plant-pathogen interaction” pathway (KO04626) was enriched in upregulated genes from leaves and downregulated genes from roots (**Figure 3B**; *p* < 0.01). This observation prompted further investigation into the differential expression patterns of ion channels responsible for encoding calcium and related proteins in these two organs. Interestingly, leaves displayed a significant upregulation of genes encoding CaM, CNGC, Ca^2+^-transport ATPase, CBP, CDPK, and CML at CH6 and CD2 time points (**Supplementary Figure S3B**). Conversely, roots subjected to cold stress exhibited downregulation trends for most genes encoding CBL, CBP, CDPK, CIPK, and CML. However, a small subset of genes encoding CNGC, Ca^2+^-transport ATPase, and CaM displayed increased expression (**Supplementary Figure S3B**). Furthermore, the expression of RBOH, a protein associated with reactive oxygen species (ROS) signaling, increased at CH6 and CD2 in leaves, with peak expression occurring at CH6 in roots (**Supplementary Figure S3B**). Additionally, genes involved in long-distance signaling, such as glutamate receptor-like channels (GLRs), reached peak expression levels at CD2 in both roots and leaves (**Supplementary Figure S3B**).

Transcription factors play critical roles in regulating the expression of genes responsive to stress conditions, including cold stress. This study investigated the expression patterns of these factors in leaves and roots of *A. annua* under cold treatment. We observed notable variations, with 760 and 500 transcription factors exhibiting differential expression in leaves and roots, respectively. These differentially expressed transcription factors were primarily associated with families such as AP2/ERF-ERF, WRKY, MYB, NAC, bHLH, and bZIP (**Supplementary Figures S4A, B**). For further analysis, we selected transcription factors displaying significant differential expression (|fold change| > 4, FDR < 0.05; **Supplementary Table S3**). Within the AP2/ERF family, several genes, including ERF054 (*Artemisia annua*_newGene_33079 and *Artemisia annua*_newGene_33080), DREB1D (CTI12_AA078540 and CTI12_AA268220), and DREB1E (CTI12_AA238990), were significantly upregulated at CH6 in leaves (**Supplementary Table S3**). For the WRKY family, we observed upregulation at CH6 in leaves for genes encoding WRKY40 (Artemisia_annua_newGene_10883, CTI12_AA259860, CTI12_AA329040, CTI12_AA449040, and CTI12_AA074230). Conversely, WRKY24 (CTI12_AA358100), WRKY54 (CTI12_AA014850), and another WRKY (CTI12_AA054210) displayed increased expression in both leaves and roots (**Supplementary Table S3**). Moreover, two members of the bHLH family, bHLH130 (CTI12_AA601930) and bHLH35 (CTI12_AA006590, CTI12_AA396330, and CTI12_AA617370), were upregulated at CH6 in leaves. Notably, we also observed upregulation of the bZIP genes CRPF1 (CTI12_AA339020 and CTI12_AA098120) and HY5 (CTI12_AA491760 and CTI12_AA604700) at CH6 in leaves (**Supplementary Table S3**). Interestingly, all differentially expressed NAC family genes were upregulated at CH6 and CD2 in leaves, while NAC2 (CTI12_AA122010) and NAC72 (CTI12_AA120720 and CTI12_AA145460) exhibited increased expression in both leaves and roots under cold stress (**Supplementary Table S3**).

### Carbon distribution and metabolism response to cold stress

**Figure 4:**
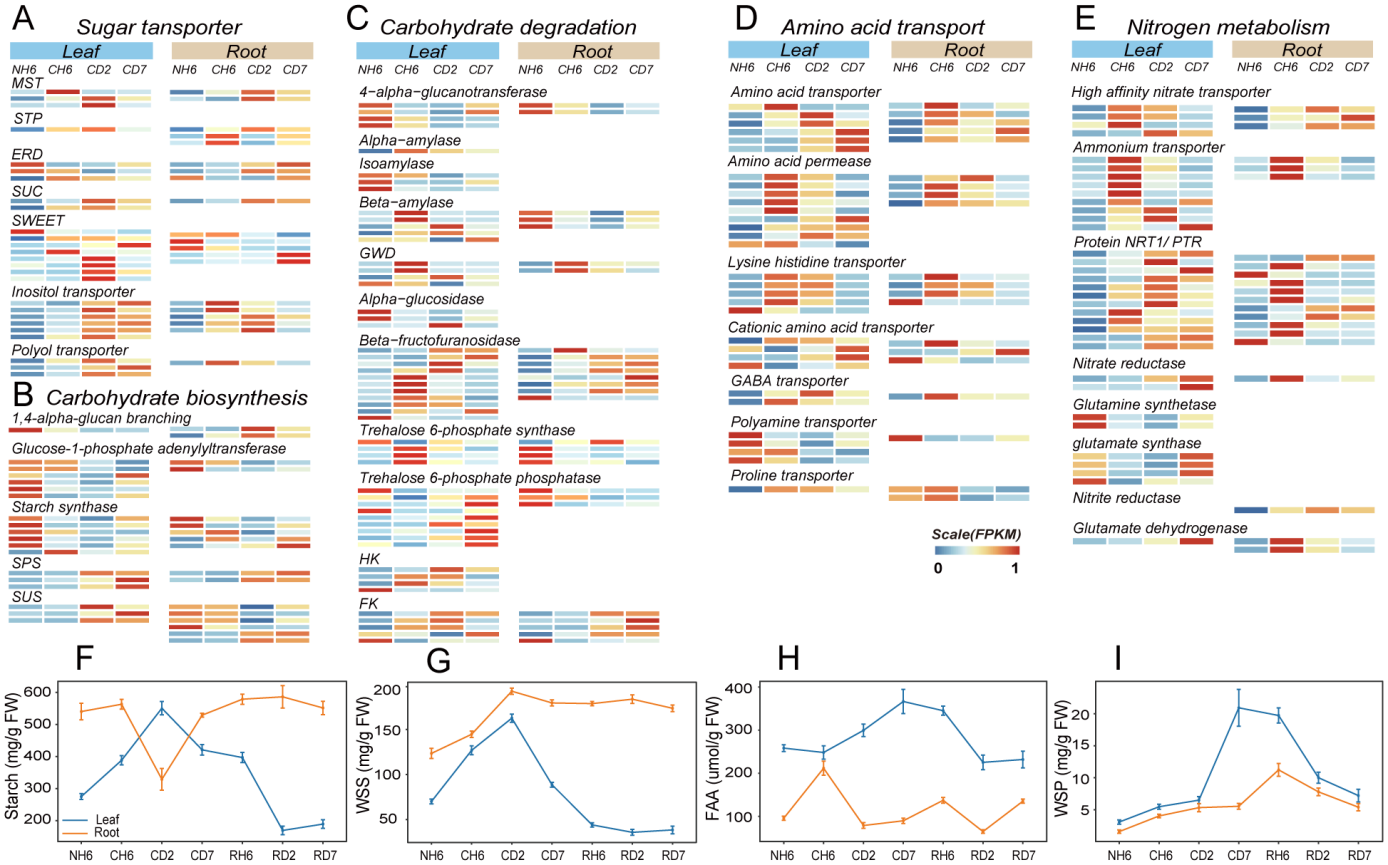
Transcriptomic and metabolite quantitative analyses revealed carbon and nitrogen metabolism and transport under cold treatment. **(A–C)** Comparison of the expression of genes related to sugar transporter **(A)**, carbohydrate biosynthesis **(B)**, and carbohydrate degradation **(C)** in leaves and roots under cold treatment [differentially expressed genes (DEGs); |fold change > 2|, false discovery rate (FDR) < 0.05). **(D, E)** Comparison of the expression of amino acid transporter **(D)** and nitrogen metabolism **(E)** genes in leaves and roots under cold treatment (DEGs; |fold change > 2|, FDR < 0.05). **(F–I)** Starch **(F)**, water-soluble sugar (WSS; **G**), free amino acid (FAA; **H**), and water-soluble protein (WSP; **I**) contents in leaves and roots throughout the experiment. The abbreviations of DW and FW represent dry weight and fresh weight, respectively.

Low temperatures can also influence the distribution of carbon sources between plant organs, leading to significant alterations in carbon metabolism (**Figure 3B**). This study investigated the dynamics of TOC to assess carbon distribution under cold stress conditions (**Supplementary Figure S5A**). The results revealed an initial increase in leaf TOC, followed by a decrease and another increase, with a peak observed at CD2. Conversely, root TOC exhibited an opposing trend. Furthermore, starch levels in leaves displayed a notable increase at CD2, following an initial rise and subsequent decline (**Figure 4F**). Starch has the primary storage form of carbohydrates in roots, and its content followed a pattern of decrease and subsequent increase, with the lowest content measured at CD2 (F). Additionally, WSS levels in both roots and leaves progressively rose, reaching a peak at CD2 (**Figure 4G**).

Following the GO enrichment analysis of DEGs categorized under “transmembrane transport” (**Figure 3A**), we investigated the enrichment of genes encoding sugar transport proteins. Sugar transport proteins, such as SUC, MST, STP, and ERD, play critical roles in carbohydrate transmembrane transport and source-to-sink movement of sucrose in the phloem (Sherson et al., 2008; Emilie and Rennie, 2009; Sharwood et al., 2020; Bavnhøj et al., 2023). Our results revealed significant upregulation of these genes in both roots and leaves upon cold exposure (**Figure 4A**). However, SWEET transporters, which facilitate bidirectional sugar transport (Chen et al., 2010), displayed divergent expression patterns. These transporters were downregulated in roots but upregulated in leaves at CD2 (**Figure 4A**). Genes encoding transporters for fixed carbon movement over long distances, such as the inositol transporter and the polyol transporter, exhibited significant upregulation in both leaves and roots at different time points (**Figure 4A**). It is well-established that plants utilize linear sugar alcohols for this long-distance transport (Pleyerová et al., 2022).

Next, we investigated gene expression patterns associated with carbohydrate biosynthesis and degradation using transcriptomic data analysis (**Figures 4B, C**). Notably, leaves exhibited significant upregulation of genes involved in starch degradation, including glucan water dikinase, alpha-amylase, and beta-amylase (**Figure 4C**). Sucrose-phosphate synthase and sucrose synthase, key enzymes in both carbohydrate biosynthesis and breakdown, were upregulated in both roots and leaves (**Figure 4C**). Additionally, beta-fructofuranosidase, which facilitates sucrose breakdown into fructose and glucose, also showed increased expression in both organs (**Figure 4C**). Given that sucrose serves as the primary transport form of carbohydrates in plants, these findings suggest enhanced metabolic activity related to both sucrose synthesis and utilization. Furthermore, key enzymes involved in fructose and glucose phosphorylation, namely, fructokinase (FK) and hexokinase (HK), play critical roles in various plant organic metabolic processes, particularly respiration (**Figure 4C**). The provided heatmap (**Supplementary Figure S6**) depicts DEGs associated with glycolysis and the tricarboxylic acid (TCA) cycle, both of which were significantly upregulated in both investigated organs. Interestingly, leaves displayed an earlier increase in respiratory activity compared to roots, suggesting a heightened energy demand in this tissue.

Finally, we examined the relationship between respiration and photosynthesis. Notably, genes associated with photosynthesis, including LHCI, LHCII, PSI, and PSII, exhibited significant downregulation at CH6 and CD2, with partial recovery observed at CD7 (**Supplementary Figures S7A, B**). Furthermore, FTSH, the gene most significantly upregulated, was associated with both “photosystem II repair” and “photoinhibition” (**Supplementary Figure S7C**), supporting its role in the proteolytic degradation of damaged D1 protein during PSII repair (Kato and Sakamoto, 2018). The functional proteins known as “early light-induced proteins (ELIPs)” displayed the highest induction rates (**Supplementary Figure S7C**). ELIPs, located in the thylakoid membrane and bound to chlorophyll *a*, play a crucial role in photoprotection (Havaux et al., 2002). Additionally, leaf anthocyanin content increased significantly at CD2 and CD7, reaching 13.45 mg/100 g FW and 12.60 mg/100 g FW, respectively (**Supplementary Figure S7D**). Previous research suggests that higher anthocyanin content is correlated with reduced chlorophyll degradation (Liu et al., 2020).

### Nitrogen distribution and metabolism response to cold stress

Cold stress disrupts nitrogen uptake and transport within plants. To investigate the impact of cold treatment on the long-distance translocation of nitrogen sources across organs, we analyzed nitrogen distribution by quantifying TN levels in both leaves and roots. Our findings revealed a significant increase in root TN content, while leaf TN content showed a significant decrease (**Supplementary Figure S5B**). Furthermore, the concentration of FAAs in leaves exhibited a marked increase at CD2 and CD7, rising by 15.9% and 42.3%, respectively (**Figure 4H**). Conversely, FAA levels in roots displayed an initial rise followed by a decline; the concentration at CH6 was 2.21-fold higher than that observed at NH6 (**Figure 4H**).

Additionally, we identified DEGs related to nitrogen metabolism, including glutamine synthase, high-affinity nitrate transporter, nitrate reductase, glutamate synthase, and glutamate dehydrogenase (**Figure 4E**). Notably, we observed the upregulation of genes encoding nitrate reductase and nitrite reductase in both organs. Our results also indicated a significant increase in both NR and NiR under low-temperature treatment, with a more pronounced increase in roots (**Supplementary Figures S5E, F**). However, the contents of ammonium nitrogen (NH_4_^+^) and nitrate nitrogen (NO_3_^−^) also increased at low temperatures (**Supplementary Figures S5C, D**). FAAs are considered the primary form of inter-organ transport (Lalonde et al., 2004). GO analysis revealed significant enrichment of DEGs in “amino acid import” and “amino acid transmembrane transport” in both leaves and roots (**Figure 3A**). In our study, genes encoding amino acid transporter, proline transporter (ProT), amino acid permease (AAPs), GABA transporter, and lysine histidine transporter (LHTs) were upregulated at CH6 and CD2 in leaves and at CH6 in roots (**Figure 4D**). In *Arabidopsis*, LHTs, ProT, and particularly AAPs have also been shown to function in long-distance transport and source-to-sink partitioning of organic nitrogen (Tegeder and Rentsch, 2010; Tegeder, 2012; Tegeder and Masclaux-Daubresse, 2017). Furthermore, some genes encoding the Protein NRT1/PTR (NPF) family exhibited upregulation in leaves and roots (**Figure 4E**). Plant NPF proteins are capable of long-distance transport of various substrates between organs, including amino acids, dipeptides, nitrates, nitrites, chloride ions, and glucosinolates (Wang et al., 2012). These results suggest that cold treatment inhibits the long-distance transport of inorganic nitrogen (NO_3_^−^, NH_4_^+^) in *A. annua*, and the roots primarily transport organic nitrogen in the form of amino acids to the above-ground parts.

**Figure 5:**
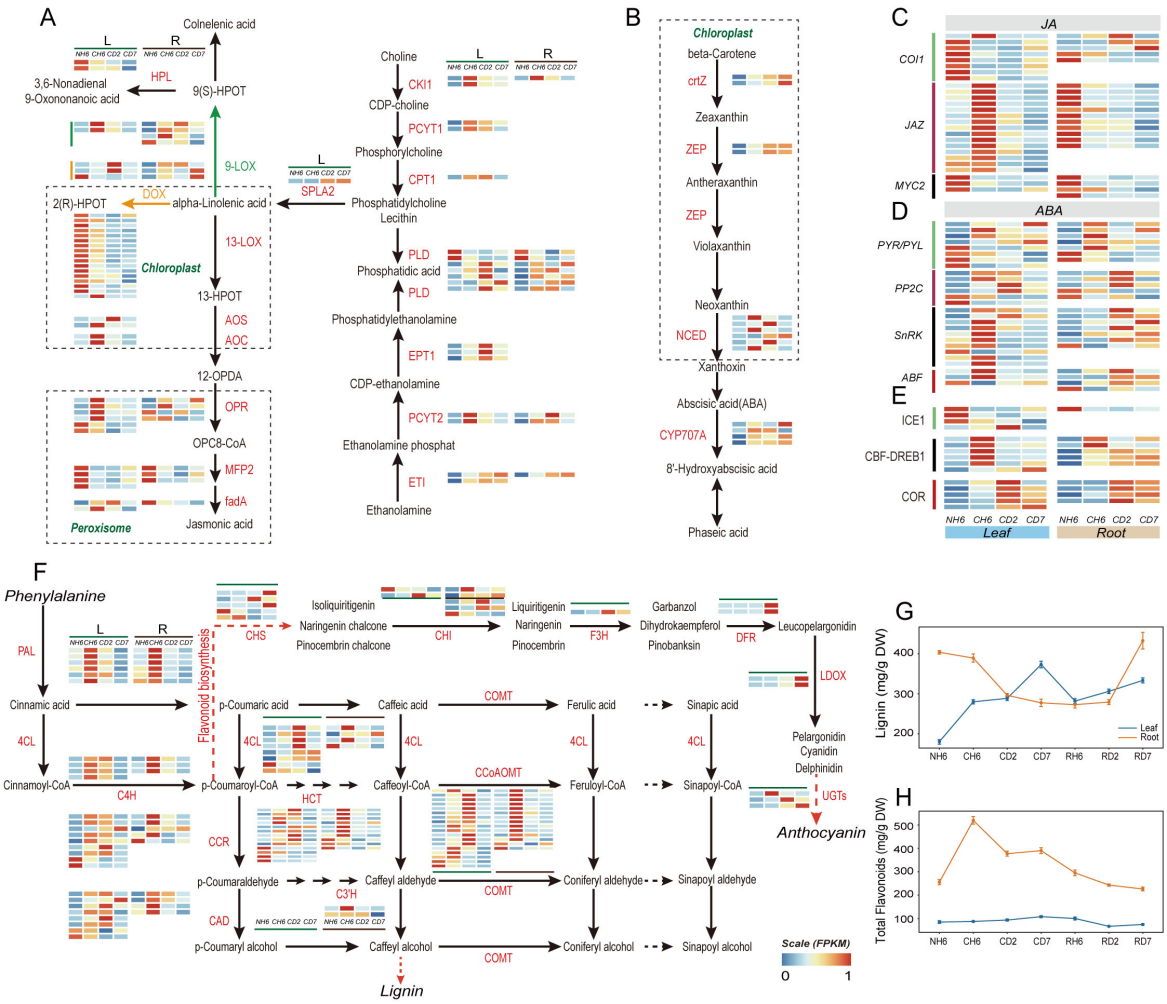
Jasmonic acid (JA) and abscisic acid (ABA) biosynthesis and signaling pathways and phenylalanine metabolism. **(A)** Pathways of “Glycerophospholipid metabolism”, “α-linolenic acid metabolism”, and “linoleic acid metabolism” in JA biosynthesis and heatmaps of differentially expressed genes (DEGs) involved in these pathways under cold treatment. **(B)** Pathway of “carotenoid biosynthesis” in ABA biosynthesis and heatmaps of DEGs involved in this pathway. **(C)** JA signaling and heatmaps of DEGs involved in this pathway under cold treatment. **(D)** ABA signaling and heatmaps of DEGs in this pathway. **(E)** Change of DEGs coding for ICE1-CBF-COR pathway. **(F)** Pathway of “phenylalanine metabolism” and heatmaps of DEGs involved in this pathway under cold treatment. **(G)** Dynamic change of lignin content in leaves and roots throughout the experiment. **(H)** Dynamic change of total flavonoid content in leaves and roots throughout the experiment. The abbreviations of DW and FW represent dry weight and fresh weight, respectively.

The transportation of amino acids from roots to leaves is crucial for establishing cold tolerance in leaves. Notably, the ICE1-CBF-COR pathway and the “Ribosome biogenesis in eukaryotes” pathway (KO00561) were previously identified in leaves (**Figures 3B**, **5E**). Additionally, we observed a gradual increase in the amount of soluble protein (WSP) in both roots and leaves, reaching its peak at RH6 (20.93 mg/g) and CD7 (20.93 mg/g), respectively (**Figure 4I**).

### Phenylalanine metabolism response to cold stress

Our analysis of KEGG Orthology (KO) pathways revealed significant enrichment in “Flavonoid biosynthesis” (KO00941), “Phenylalanine metabolism” (KO00360), and “Phenylpropanoid biosynthesis” (KO00940) in both leaves and roots (**Figure 3B**). Notably, both lignin and flavonoid synthesis shared a common upstream pathway involving the conversion of phenylalanine to *p*-coumaroyl-CoA. We observed significant upregulation of PAL, 4CL, and C4H genes at CH6 and CD2 in leaves, with the highest expression levels detected at CH6 in roots (**Figure 4A**). The presence of *p*-coumaric acid and caffeic acid in *A. annua* suggests the accumulation of sufficient substrates for these downstream pathways, potentially enhancing the plant’s defense mechanisms against cold stress-induced reactive oxygen species.

Lignin enhances cell wall elasticity, increases adaptability to ice crystals, and reduces cell damage caused by dehydration during cold acclimation. Notably, our findings showed a rapid induction of lignin content in leaves at CH6, CD2, and CD7, reaching 289.89 mg/g FW, 288.51 mg/g FW, and 373.16 mg/g FW, respectively (**Figure 5G**). Conversely, roots exhibited a marked reduction in lignin content, especially at CD2 and CD7. Lignin content in roots at these time points was 389.28 mg/g FW, 296.13 mg/g FW, and 277.34 mg/g FW, respectively (**Figure 5G**). However, roots exhibited a fast accumulation of flavonoids at CH6, CD2, and CD7, with levels of 519.75 µg/g FW, 377.41 µg/g FW, and 390.49 µg/g FW, respectively (**Figure 5H**). According to GO analysis, the “phenylpropanoid biosynthetic process” was found to be significantly enriched in the upregulated genes at CH6 and CD2 in leaves (**Figure 3A**), including genes responsible for Dirigent proteins (**Supplementary Table S5**). These genes play a role in synthesizing lignin polymers from lignin monomers (Yi-Qun et al., 2023).

### ABA, JA, and cell membrane system-related genes respond to cold stress

Our previous observations (**Figure 3B**) revealed significant enrichment of DEGs in both leaves and roots within pathways related to “linoleic acid metabolism” (KO00591), “alpha-linolenic acid metabolism” (KO00592), and “Biosynthesis of unsaturated fatty acids” (KO01040). Genes encoding Delta-12 desaturase (SAD) and fatty acid desaturase (FAD) were upregulated in both leaves and roots (**Supplementary Table S4**). Alpha-linolenic acid and linoleic acid, polyunsaturated fatty acids (PUFAs), and common substrates for lipoxygenase (LOX) contribute to membrane fluidity (Porta and Rocha-Sosa, 2002; Upchurch, 2008). Interestingly, most lipoxygenase-related genes involved in JA biosynthesis were downregulated in leaves at CD2. Key genes like AOS, AOC, and OPR in the JA synthesis pathway were significantly upregulated at CH6 in leaves (**Figure 5A**). This suggests a potential for transient JA production followed by a decline at later stages. JAZ proteins are integral components of the JA signaling pathway, and their expression is induced in response to JA treatment (Chini et al., 2007; Thines et al., 2007). Consistently, genes encoding JAZ proteins were upregulated at CH6 in leaves (**Figure 5D**), suggesting a potential early increase in JA signaling. Furthermore, the “Carotenoid biosynthesis” pathway, implicated in both the biosynthesis and breakdown of ABA, was enriched in leaves (**Figure 3B**). Notably, the expressions of ZEP, NCED, and CHY-β, all key enzymes in ABA biosynthesis, were upregulated under cold treatment in leaves (**Figure 5B**). Additionally, in ABA signaling, majority of genes were upregulated at CH6 in leaves, including genes encoding for PP2C, SnRK and ABF (**Figure 5C**). Based on this evidence, we posit that the content of ABA likely increased in leaves during cold stress in *A. annua*.

Following biosynthesis in leaves, ABA and JA require long-distance transport to other organs (Manzi et al., 2015; Anfang and Shani, 2021). GO analysis revealed significant enrichment of the “Drug transmembrane transport” (GO:0006855) pathway in upregulated genes at CD2 and CD7 in leaves (**Figure 3A**). Notably, these enriched genes encode ABCG transporters and Detoxification Efflux Carriers (DTX)/Multidrug and Toxic Compound Extrusion (MATE) family transporters (**Supplementary Table S5**), both of which are implicated in the long-distance transport of ABA (Kuromori et al., 2010; Zhang et al., 2014; Kang et al., 2015; Yuqin et al., 2021; Peng et al., 2021b; Ying et al., 2023).

### Circadian rhythm in *A. annua* under cold and recovery treatment

**Figure 6:**
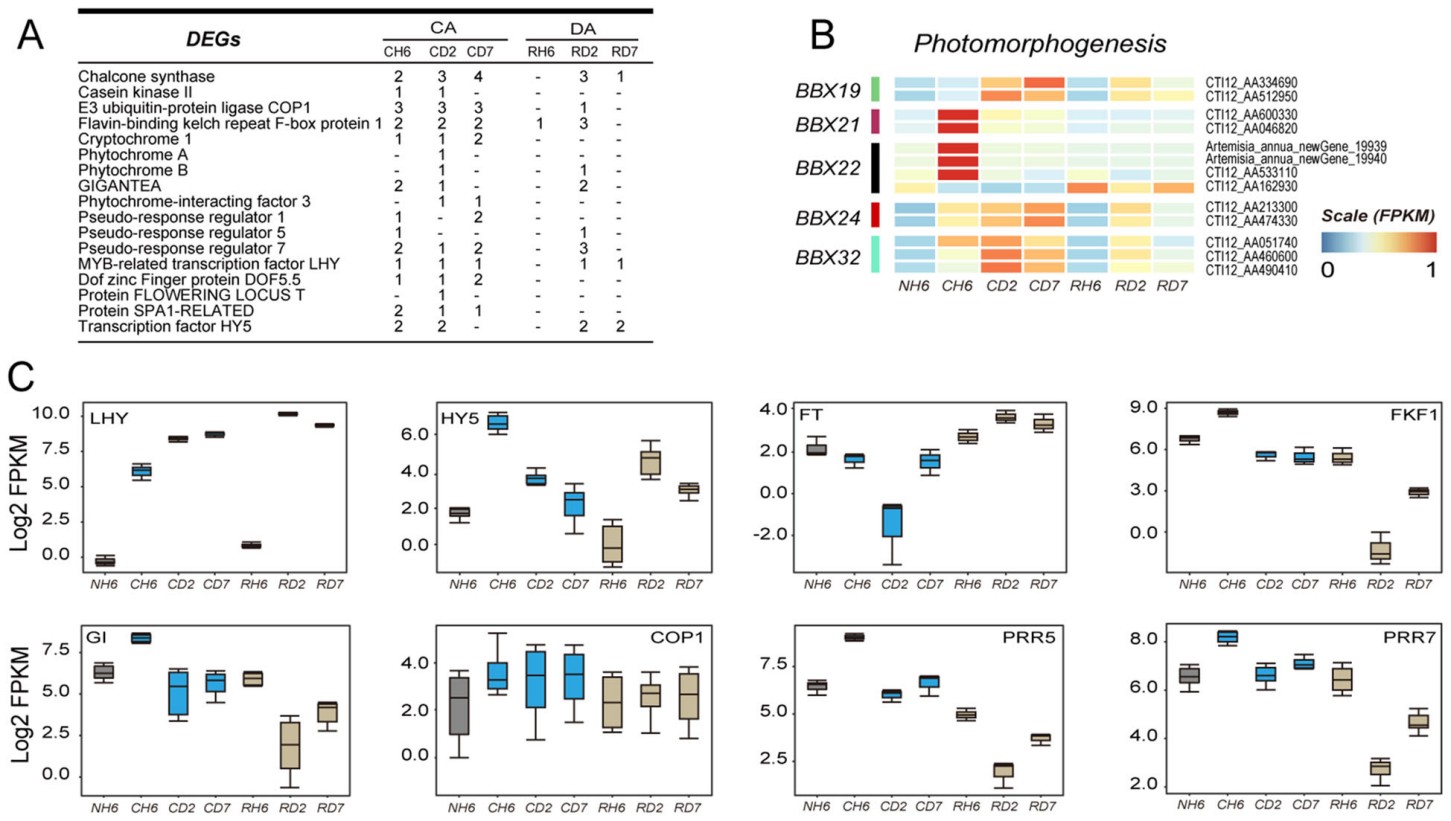
Transcriptomic data analysis of the changes in circadian rhythm-related genes. **(A)** Number of differentially expressed genes (DEGs) annotated to the “circadian rhythm-plant” pathway in each group throughout the experiment. **(B)** Heatmap showing DEGs enriched in “photomorphogenesis” according to Gene Ontology (GO) analysis. **(C)** Boxes showing the expression of DEGs related to “circadian rhythm—plant”.

KEGG and GO analyses identified significant enrichment of genes related to “circadian rhythm-plant” and “photomorphogenesis” in leaves under cold and recovery treatment (**Figures 3A, B**; **Supplementary Tables S7**, **S8**). Specifically, DEGs encoding BBX19, BBX21, BBX22, BBX24, and BBX32 were identified within the “photomorphogenesis” pathway (GO:0009640) (**Figure 6B**). In our study, BBX21 and BBX22 were upregulated at CH6, whereas BBX19, BBX24, and BBX32 were upregulated under both cold and subsequent recovery treatment (**Figure 6B**). Moreover, DEGs involved in circadian rhythms primarily included Flavin-binding kelch repeat F-box protein 1 (FKF1), GIGANTEA (GI), pseudo-response regulator (PPR), transcription factor LHY, and transcription factor HY5 (**Figure 6A**). The genes encoding HY5 were most significantly upregulated throughout the experiment (**Figure 6C**). Notably, the expression pattern of LHY closely mirrored that of HY5, showing strong induction (**Figure 6C**). Furthermore, other circadian rhythm-related genes, including COP1, FKF1, GI, FT (FLOWERING LOCUS T protein), PRR5, and PRR7, exhibited differential expression patterns (**Figure 6C**). These findings suggest that circadian rhythms may play a role in integrating temperature changes and the plant’s internal rhythms, thereby influencing plant defense mechanisms and growth.

### WGCNA of transcriptome samples

**Figure 7:**
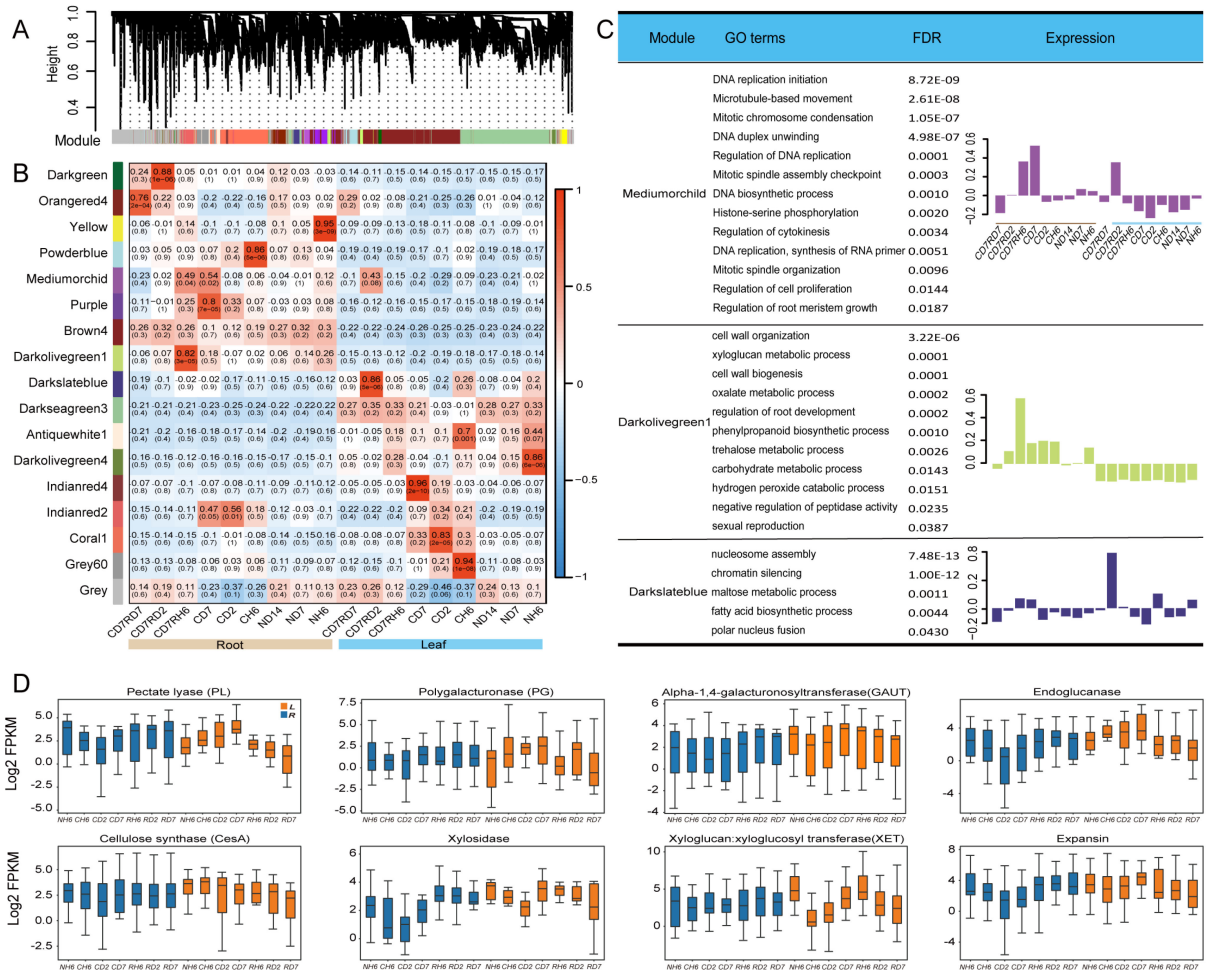
Cell wall synthesis and weighted gene co-expression network analysis (WGCNA)of RNA sequencing (RNA-seq). **(A)** Hierarchical cluster tree of the WGCNA showing 17 modules of co-expression genes. Each transcript is represented by a leaf in the tree, and each of the 17 modules is represented by a major tree branch. The following panel describes the module in the specified color, such as “Darkgreen” and “Gray”. **(B)** Significance of the correlation between the modules and eigenvalues of the samples. The left panel provides color mapping for the 17 modules. The color code on the right shows the module feature correlation from 1 (Darkgreen) to 17 (Gray). **(C)** For the WGCNA modules in Mediumorchild and Darkolivegreen1, we summarized Gene Ontology (GO) classifications [false discovery rate (FDR) < 0.05]. **(D)** Boxes showing comparisons of the expression of differentially expressed genes (DEGs) related to “cell wall” terms.

To gain insights into co-expressed genes rather than focusing on individual genes, a global weighted gene co-expression network analysis (WGCNA) was performed on all transcriptome samples (Langfelder and Horvath, 2008). This analysis identified 17 modules, each containing genes with similar expression trends (**Figure 7C**, **Supplementary Table S6**). Genes within each module exhibited coordinated expression patterns (**Figure 7A**).

Two modules captured our particular attention. The Mediumorchild module exhibited a strong positive correlation with CD7 (0.52, 0.002) and RH6 (0.49, 0.004) in roots and RD2 in leaves (0.43, 0.06; **Figure 7B**). GO analysis revealed significant enrichment of genes within this module in processes like “DNA replication initiation”, “Mitotic chromosome condensation”, “Regulation of DNA replication”, “Mitotic spindle assembly checkpoint”, “DNA biosynthetic process”, and “Regulation of cytokinesis” (**Figure 7C**). These enrichments align well with the observed growth of *A. annua*. Additionally, the Darkolivegreen1 module (Roots, 0.82) displayed enrichment in “xyloglucan metabolic process” and “cell wall organization/biogenesis” (**Figure 7C**). Notably, cell wall stress and turgor pressure are potential factors influencing cell expansion and growth (Cosgrove, 2022). Focusing on cell wall-related DEGs in leaves and roots, we investigated expansins and xyloglucan endotransglucosylase/hydrolase (XETs). Expansin-encoding genes exhibited downregulation in leaves under cold treatment (**Figure 7D**). While XETs are likely involved in cell expansion (Braam, 1999), more conclusive evidence is needed. However, the XET function may be relevant in lateral root primordia, where cell wall loosening is crucial (Vissenberg et al., 2001). In our study, multiple XTHs showed significant downregulation in roots, suggesting a potential role in facilitating cell wall loosening necessary for lateral root emergence (**Figure 7D**). We propose that the mechanical force generated by pericycle growth may induce XTH expression at these sites. XET action could then aid in either wall loosening required for cellular emergence or wall repair as the nascent lateral root pushes through the cortex and epidermis.

Additionally, our transcriptomic data revealed significant variations in the expression of genes associated with cell wall metabolism between leaves and roots (**Figure 7D**). These genes included polygalacturonase, pectate lyase, endoglucanase, xylosidase, alpha-1,4-galacturonosyltransferase, and cellulose synthase. Based on these findings, we hypothesized that cold stress-induced differential regulation of cell wall components potentially leads to the thickening of leaf cell walls and a concomitant decrease in root cell wall thickness.

Further analysis revealed that many DEGs are implicated in the biosynthesis and signaling pathways of auxin, cytokinin, and ethylene. YUC, IPT, and LOG, the key genes involved in auxin and cytokinin biosynthesis, were significantly upregulated in both leaves and roots (**Supplementary Figure S8A**). Conversely, genes encoding AUX1 and CRE1 exhibited significant downregulation in leaves (**Supplementary Figures S8B, E**), suggesting a suppression of auxin and cytokinin transport. GO analysis identified the downregulation of genes associated with “plasmodesmata-mediated intercellular transport” (GO:0010497, FDR = 0.02) in roots at CD7 (**Figure 3A**). Previous research has found that plasmodesmata-mediated transport in *Arabidopsis* supports auxin channeling (Gao et al., 2020). This downregulation may have an impact on the spatial distribution of auxin. Additionally, many crucial genes involved in ethylene production and signaling were upregulated in roots at CH6 (**Supplementary Figures S8A, E**). Prior research has established that auxin, cytokinin, and ethylene can control the initiation of lateral root primordia (Muday et al., 2012).

## Discussion

**Figure 8:**
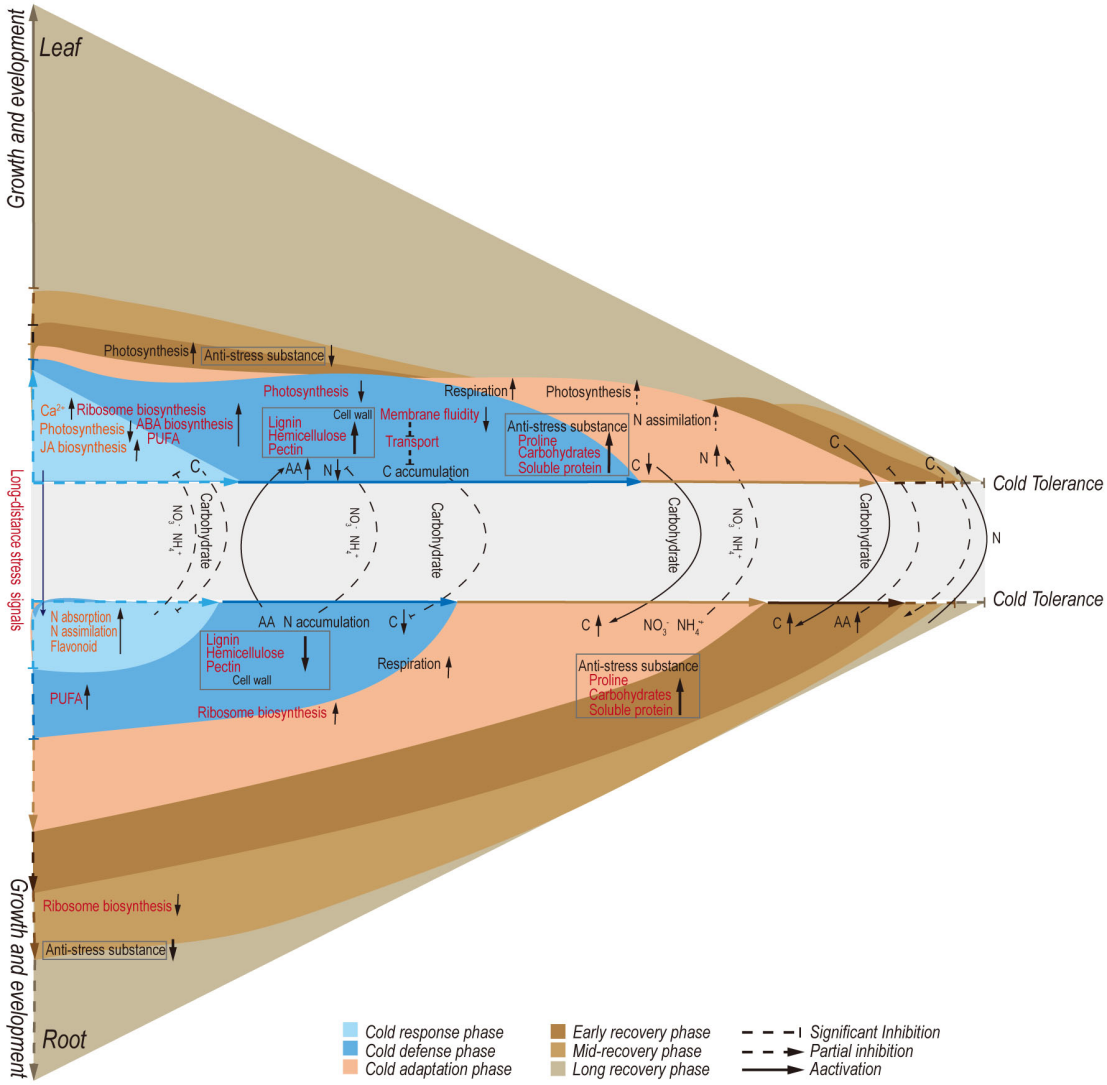
Proposed model for metabolic regulations involved in defense and growth strategies of *Artemisia annua* under cold adaptation and de-adaptation.

The increasing frequency of extreme weather events has elevated climate to the primary factor influencing plant growth and survival. Plant responses to cold stress are known to involve changes in both transcription and metabolism. This study divided the cold adaptation and de-adaptation of *A. annua* into five distinct stages: initial cold response, defense establishment, cold adaptation, early recovery, and late recovery stage (**Figure 8**). Through transcriptome dynamics and metabolite quantitative analysis, we found that each stage exhibits unique characteristics in both leaves and roots. Furthermore, the distribution of TN and TOC illuminates the relationships between these stages.

### Differences in the main regulatory factors and mechanisms of leaves and roots under cold stress

In the initial cold response, leaves exhibited a faster transcriptome response compared to roots upon exposure to cold (**Figure 8**). The main leaf characteristics were enhanced activity in signal transduction and transcriptional regulation mechanisms. Several studies have reported that Ca^2+^ functions as a secondary messenger in response to cold stress, interacting with Ca^2+^-binding proteins like calmodulin/CaM-like proteins (CaM/CML), Ca^2+^ dependent protein kinases (CDPK), and calcineurin B-like proteins/calcineurin B-like protein-interacting protein kinases (CBL/CIPK) (Guo et al., 2018; Zhao et al., 2019). In our study, the abundance of genes encoding Ca^2+^ channel proteins and decoders was rapidly induced in leaves (**Supplementary Figures S3A, B**), leading to an elevation in intracellular Ca^2+^ levels and subsequent activation of downstream signaling pathways. In contrast, roots showed a significant decline in the number of these genes at this time point (**Supplementary Figure S3B**). Specifically, genes encoding Ca^2+^ channel proteins, cyclic nucleotide-gated ion channel (CNGC), and Ca^2+^-transporting ATPase were upregulated at CH6 and CD2 in leaves. Studies in rice have shown that overexpression of CNGC genes enhances chilling tolerance (Cui et al., 2020) and that overexpression of Ca^2+^-transporting ATPase genes confers cold tolerance in *tobacco* (Kamrul Huda et al., 2014). Moreover, genes encoding GLRs were upregulated in both leaves and roots at CH6 and CD2 (**Supplementary Figure S3B**). According to recent studies, GLRs are essential for the transmission of calcium waves, enabling long-distance signaling between organs (Annalisa et al., 2022). In conclusion, the differential expression of these genes between leaves and roots may suggest that *A. annua* prioritizes the activation of cold defense mechanisms in leaves, while roots maintain stricter control over intracellular Ca^2+^ concentrations to mitigate intense cold responses.

In downstream transcriptional regulation within the AP2/ERF-ERF family, DREB1D (CTI12_AA078540), DREB1E (CTI12_AA238990), and ERF054 (Artemisia_annua_newGene_33079 and Artemisia_annua_newGene_33080) were specifically upregulated at CH6 in leaves (**Supplementary Table S3**). DREB1, a regulatory factor in the classical ICE-CBF-COR pathway (Shi et al., 2018), exhibited rapid induction in leaves under low-temperature conditions. ERF54 in *Rosa multiflora* has been linked to cold tolerance via the DREB/COR signaling pathway (Chen et al., 2024). Notably, DREB1B (CTI12_AA111400) showed significant downregulation in roots after cold treatment (**Supplementary Table S3**). Furthermore, bZIP-type transcription factors are crucial in regulating the expression of ABA-responsive genes by binding to specific elements such as the G-box or ABA-responsive elements (ABREs) in their promoters (Ma et al., 2023). ABREs are identified as major *cis*-acting elements in CORs (Huang et al., 2011). ABA, a well-studied hormone involved in cold stress regulation, rapidly initiates cold defense mechanisms through increased abscisic acid synthesis. In our study, key genes ZEP and NCED in the ABA synthesis pathway were significantly upregulated in leaves at low temperatures (**Figure 5B**), suggesting that leaves under cold stress had higher ABA content. However, within the JA signal pathway, MYC2, a member of the bHLH family, interacts with ICE1 to enhance the transcription of CBF genes (Wang et al., 2018). Notably, the genes encoding MYC2 showed significant downregulation in both leaves and roots, and the gene for LOX, an upstream component of JA synthesis, was markedly downregulated (**Figures 5A, D**). Moreover, EIN3 within the ethylene signaling pathway can directly bind to the CBF promoter to inhibit its expression (Jiang et al., 2017). In this study, genes encoding EIN3 were upregulated both in leaves and roots (**Supplementary Figure S8E**).

MYB36, a member of the MYB family, influences the expression of Dirigent proteins (DPs) and contributes to the polymerization of lignin monomers into lignin co-polymers (Yi-Qun et al., 2023). Interestingly, no differential expression of genes encoding MYB36 was found, despite the significant upregulation of DPs in leaves (**Supplementary Table S5**). This suggests that underlying transcription factors may regulate DP expression. Previous research has demonstrated that common plant regulatory factor 1 (CPRF1) within the bHLH family can interact with ACGT elements of the CHS promoter to negatively regulate the expression of CHS (Michael et al., 1994). In this study, two CPRF1-encoding genes were discovered to be upregulated at CH6 and CD2 in leaves (**Supplementary Table S3**). This upregulation of CPRF1 may reroute the phenylalanine metabolism pathway toward lignin synthesis (**Figures 5G**, **4H**). Additionally, ERF1B of the AP2/ERF-ERF family has been linked to regulating sugar-induced flavonoid biosynthesis (Lv et al., 2022). The genes encoding ERF1B (CTI12_AA061730 and CTI12_AA548660) were found to be specifically upregulated at CH6 in roots in our investigation (**Supplementary Table S3**). This finding aligns with the observation that the phenylalanine metabolism pathway in roots predominantly reroutes to flavonoid production (**Figures 5G, H**).

Cold stress can directly inhibit metabolic reactions and contribute to developing osmotic and oxidative stresses. Cold stress can directly inhibit metabolic reactions and contribute to developing osmotic and oxidative stresses. Three genes (CTI12_AA006590, CTI12_AA396330, and CTI12_AA617370) encoding bHLH35 and one gene (CTI12_AA077530) encoding bHLH130 were specifically upregulated at CH6 in leaves. Previous research in *tobacco* has shown that bHLH130 functions as a positive regulator of water stress responses by influencing stomatal closure and ROS-scavenging (Zhao et al., 2020). Similarly, bHLH35 has been identified as a positive regulator of drought stress responses in *Arabidopsis* by controlling stomatal density, aperture, photosynthesis, and growth (Dong et al., 2014). Additionally, members of the WRKY family, WRKY54 and WRKY75, have been linked to proline accumulation and the activation of genes that respond to osmotic stress (Li et al., 2013). In this study, WRKY54 (CTI12_AA014850) and WRKY75 (CTI12_AA358100) encoding genes were found to be upregulated at CH6 and CD2 in both leaves and roots (**Supplementary Table S3**). Interestingly, the majority of NAC family transcription factors were specifically upregulated in leaves under cold stress (**Supplementary Table S3**), mainly including NAC68, NAC83, and NAC90. Moreover, NAC2 and NAC72 (CTI12_AA122010, CTI12_AA120720, and CTI12_AA145460) were found to be upregulated in both organs (**Supplementary Table S3**). Although prior research has demonstrated that cold stress induces many NAC transcription factors (Diao et al., 2020), more investigation is necessary to clarify their functional mechanisms and the causes of their organ-specific reactions.

In brief, regulating plant responses under cold stress is a complex process. In *A. annua*, the intricate hormone signaling crosstalk of the ICE-CBF-COR pathway was first triggered in leaves, and additionally, leaves exhibited regulation through a multitude of other pathways. In contrast, the transcriptional control in root tissues was simpler. We hypothesized that specific transcription factors may impact the phenylalanine pathways connecting these two organs. Nevertheless, the functionality of each identified gene requires validation through diverse methodologies. Consequently, further research is crucial to fully unravel the cold stress response mechanisms in *A. annua*.

### The long transport of carbohydrates under low temperatures affects the carbon metabolism of different organs

The dynamic flow and concentration changes of carbonaceous compounds between organs under low temperatures demand further investigation. Our study revealed that the transport of carbohydrates from leaves to roots undergoes a process of stopping and restarting under cold stress (**Figure 8**). Previous research has shown that phloem transport of carbohydrates temporarily halts at low temperatures. This cessation is localized and transient, with phloem transport resuming even if the plant remains cold (Peuke et al., 2005). However, chloroplasts suffer damage during initial exposure to low temperatures, which may be the primary cause of starch accumulation in leaves and the reduction of photosynthesis (Martin et al., 2002). This disruption affects the balance between the production and export of chloroplast photosynthetic products. The dynamic changes in gene expression associated with photosynthesis (**Supplementary Figures S7A, B**), sugar transport proteins (**Figure 4A**), and membrane systems (**Figure 5A**), coupled with the alterations in net photosynthetic rates (**Figure 1B**), suggest that removing excess photoassimilates from source chloroplasts is vital for preserving their functionality and structural integrity. This mechanism presumably plays a role in cold adaptation.

Photosynthesis and respiration are not only sensitive to temperature fluctuations but also interdependent (Wang et al., 2020). Respiration depends on the products of photosynthesis, while photosynthesis relies on the compounds of respiration. This is particularly important for overwintering plants, where the widely accepted theory is that respiration rates decrease during winter to accumulate carbohydrates. These carbohydrates act as cell osmolytes or cryoprotectors, helping plants survive freezing temperatures. In our study, WSS accumulated under cold stress (**Figure 8**). Most DEGs in the TCA cycle and glycolysis showed the highest expression at CD2 (**Supplementary Figure S6**). Similar observations of increased respiration during cold stress have been reported in *paper mulberry* and other woody species (Peng et al., 2015; Silva et al., 2015). Previous research has suggested that the energy provided by respiration can help repair D1 proteins and maintain the proton gradient across the thylakoid membrane for the xanthophyll cycle (Hurry et al., 1995). Moreover, carbohydrate metabolism also changed in roots. An enhancement of respiration occurred at a later stage compared to leaves (**Figure 3B**). Significant downregulation was observed in genes linked to cell wall synthesis, while significant upregulation was observed in genes related to pectin and cellulose degradation (**Figure 7D**). These changes may be caused by the inhibition of carbohydrate transport from above-ground leaves to underground roots during early cold treatment, resulting in limited utilization of carbon sources. In addition, the growth rate of roots is also associated with sugar content (Scheible et al., 1997; Hermans et al., 2006). Our data indicated an increased carbon flow to the roots under extended low-temperature exposure, with accumulation of TOC and starch (**Figure 4F**; **Supplementary Figure S5A**), facilitating primary root growth even in cold environments (**Figure 1C**).

In addition to the biosynthesis and metabolism of carbohydrates, a significant portion of plants’ carbon flow may be directed through the shikimate pathway, leading to the production of diverse metabolites, including aromatic amino acids (Yokoyama et al., 2021). Notably, the downstream products of phenylalanine metabolism differed significantly between leaves and roots (**Figures 5G, H**). Lignin, a structural component deposited in plant cell walls, enhances resistance to cold stress damage (Le Gall et al., 2015). In our study, we observed a significant increase in the lignin content of leaves, likely contributing to preserving their morphological structure under cold stress. Under abiotic stress conditions, flavonoids are known to accumulate and play a crucial role in stress resistance. These compounds act as antioxidants, scavenging ROS, and can directly protect proteins and membranes from freezing damage (Agati et al., 2012; Nakabayashi et al., 2013). Our results showed a significant accumulation of flavonoids in roots at CH6 (**Figure 5H**), potentially contributing to the enhanced cold tolerance observed in roots at the early stages of cold stress. This differential metabolite accumulation could be one of the reasons behind the delayed cold response observed in roots compared to leaves. Indeed, the flow of phenylalanine metabolism between the two organs is impacted by the cessation and restart of long-distance carbon transport under cold stress. Understanding the precise mechanisms behind this regulation remains a challenge and represents a promising area for further research in plant abiotic stress responses.

### The long-distance transport of nitrogen determines the cold adaptation of above-ground leaves

Photosynthesis and respiration rates exhibit a correlation with nitrogen concentration (Atkin et al., 2015). Consequently, the absorption and transportation of nutrients, with nitrogen uptake being particularly sensitive, can be affected by low temperatures, influencing plant growth (Soualiou et al., 2023). In our study, we observed a significant increase in NR and NiR activities under low-temperature treatment (**Figures 5E, F**). Additionally, a decrease in TN and an increase in NO_3_^−^ and NH_4_^+^ in leaves were noted (**Supplementary Figures S5B–D**), indicating that the nitrogen assimilation ability of leaves was insufficient to meet the demands under cold stress.

The distribution of nitrogen sources among different plant organs was a key factor contributing to the improved cold tolerance observed in leaves (**Figure 8**). Our study showed a discernible decrease in TN content in leaves and a corresponding accumulation in roots (**Supplementary Figure S5B**). FAA and NO_3_^−^ are the primary forms of nitrogen transported across various organs through the xylem and phloem pathways (Lin et al., 2008; Okumoto and Pilot, 2011). Low temperatures have been shown not to impede the phloem sap’s conveyance of inorganic solutes or amino acids (Peuke et al., 2005). In certain species like rye *Secale cereale* and *Brassica napus*, cold stress triggers a phloem-based redistribution of inorganic nitrogen from above-ground parts to roots while simultaneously inhibiting xylem-mediated nitrogen translocation to shoots (Laine and Boucaud, 1994). In our study, the accumulation of TN in roots may be attributed to the reduced efficiency of nitrogen assimilation in leaves, as evidenced by the downregulation of genes related to nitrogen metabolism at CH6 and CD2 (**Figure 4E**). Combined with the dynamic changes observed in FAA content (**Figure 4H**), these findings suggest that the transport of amino acids from roots to above-ground parts emerged as the primary mechanism for sustaining nitrogen consumption in leaves during cold stress. Furthermore, the inter-organ nitrogen flow also impacts carbohydrate distribution, with nitrogen deficiency leading to carbohydrate accumulation in leaves and increased carbon allocation to roots (Marschner and Cakmak, 1996; Sehoefs et al., 2001; Remans et al., 2006).

### Circadian rhythms play a key role in adaptation and de-adaptation

Plants synchronize their internal rhythm (physiology and metabolism) with external rhythms by integrating changes in environmental conditions, such as light and temperature. Our research demonstrated the critical role of the circadian rhythm in facilitating plants’ ability to adapt and de-adapt (**Figure 3B**; **Supplementary Table S7**).

Disruption of the circadian clock has been identified as a major factor contributing to significant variations in the transcriptome response to cold stress (Bieniawska et al., 2008). The circadian clock acts as a regulatory mechanism for activating cold-responsive genes, such as CBFs (Fowler et al., 2005). In *Arabidopsis*, key circadian oscillators like CIRCADIAN CLOCK-ASSOCIATED 1 (CCA1) and LATE ELONGATED HYPOCOTYL (LHY) play a crucial role in regulating the expression of cold-responsive genes like CBF (Kidokoro et al., 2017). Our study observed a notable upregulation of LHY in leaves subjected to cold treatment (**Figure 6C**), indicating its pivotal role in cold adaptation. For instance, genes encoding HY5, a transcription factor of the basic leucine zipper (bZIP) family, were strongly induced in leaves under cold conditions (**Figure 6C**, **Supplementary Table S3**). HY5 acts as a central regulator, influencing the expression of numerous genes involved in cold responses (Gangappa and Botto, 2016). In *Arabidopsis*, it induces the expression of 10% of all cold-inducing genes (Li et al., 2021). In *tomato*, leaves with relatively high expression of SlHY5 have stronger freeze resistance, and SlHY5 directly binds to the promoter of SlNCED6, a key ABA synthesis enzyme, and induces ABA biosynthesis (Wang et al., 2019). Additionally, HY5 has been found to integrate light and cold signaling to enhance plant survival under low temperatures. Our work also revealed the upregulation of genes from the BBX family, particularly BBX21 and BBX22, associated with photomorphogenesis (**Figure 6B**), which have been shown to interact with HY5 to modulate gene expression (Datta et al., 2008; Job et al., 2018). However, BBX24, BBX25, and BBX28 repress the expression of HY5 (Gangappa et al., 2013; Lin et al., 2018). In our study, BBX24, BBX19, and BBX32 also had similar expression patterns (**Figure 6B**), suggesting that BBX19 and BBX32 may act as potential negative regulators of HY5 to prevent excessive cold responses. While there is no direct evidence to prove whether HY5 can induce ABA accumulation in *A. annua*, we speculate that HY5 is likely involved in the rapid initiation of the cold response.

Circadian rhythm also plays an important role in growth and development after de-adaptation. In *Arabidopsis*, HY5, along with its partner HYH, positively regulates the expression of two key genes in nitrogen signaling, NITRATE REDUCTASE 2 and NITRITE REDUCTASE 1 (Lillo, 2008; Yanagisawa, 2014; Huang et al., 2015), and these genes encode enzymes responsible for converting nitrate to nitrite and nitrite to ammonium, respectively, within the cytosol. HY5 has been shown to relocate to roots and activate its own expression there, promoting nitrate uptake by activating the nitrate transporter NRT2.1 gene. It also promotes carbon assimilation and translocation (Chen et al., 2016). In our study, the genes encoding HY5 and LHY remained strongly upregulated at RD2 and RD7 (**Figure 6C**). Notably, NR and NiR activities increased significantly under cold stress and recovery treatment (**Supplementary Figures S5E, F**). Combined with the dynamic changes observed in TN and TOC content (**Figure 8**), we suggest that HY5 is involved in adjusting the carbon–nitrogen balance between organs during both cold stress and the de-adaptation period. FKF1, GI, PRR5, and PRR7 were downregulated especially at RD2 (**Figure 6C**). These genes are primarily involved in plant development. In *Arabidopsis*, FKF1 and GI proteins positively regulate the transcription of the zinc finger protein CONSTANS (CO), which regulates the flowering pathway (Imaizumi and Kay, 2006; Sawa et al., 2007). Additionally, Norihito Nakamichi et al. reported that CCA1/LHY repress CO through GIGANTEA (GI), while PRR9, PRR7, and PRR5 activate CO predominantly by repressing CYCLING DOF FACTOR1 (CDF1), a DNA-binding transcriptional repressor (Nakamichi et al., 2007). In our study, we observed phenotypic changes after 2 days of recovery treatment (**Figures 1A, C**), with a higher leaf growth rate compared to periods of cold stress and early de-adaptation. Therefore, we hypothesize that the circadian rhythm is essential for the recovery of growth and development in *A. annua* during de-adaptation.

### *A. annua* regulates defense and growth strategies during cold adaptation and de-adaptation

To survive low temperatures, plants must develop cold tolerance (Hassan et al., 2021). Our model suggests that cold stress triggers a rapid transcriptome response in leaves, whereas the response in roots is more moderate (**Figure 8**). For example, leaves exhibit a more intricate and rapid activation of Ca^2+^ signaling and transcriptional regulatory mechanisms. This early response leads to the swift establishment of cold-resistant mechanisms in leaves, such as enhanced respiration and accumulation of stress-protective compounds (**Figure 8**). Several factors may explain these observations. 1) Roots are buffered from temperature fluctuations by the insulating soil, while cold stress in leaves often coincides with oxidative and osmotic stress. 2) Plants strategically prioritize cold response allocation, directing limited resources toward organs most exposed to stress (leaves). 3) The root system may possess a basal level of inherent stress resistance. 4) The rapid accumulation of flavonoids in roots under cold stress could further enhance their stress tolerance.

*A. annua* exhibits distinct growth and development strategies in different organs under cold stress. While above-ground growth ceases completely (**Figure 1C**), primary root elongation persists at a reduced rate. This differential response is potentially reflected in the WGCNA, where genes associated with growth and development within the “Mediumorchid” module show high expression during the adaptation stage in roots (**Figures 7B, C**). Furthermore, *A. annua*’s defense and growth strategy hinges on the coordinated metabolism and transport of carbon and nitrogen across organs during cold stress. Changes in lignin content and cell wall-related genes suggest thicker cell walls in leaves compared to roots (**Figure 5G**). Similar to observations in *soybean*, lignin accumulation in leaves may contribute to reduced cell wall elongation (Blazquez et al., 2013). While the thinning of root cell walls could potentially affect nutrient uptake, further research is needed to substantiate this hypothesis. Conversely, nitrogen transport from roots to leaves in the form of amino acids could bolster cold tolerance in leaves (**Figure 8**). Additionally, the gradual restoration of long-distance carbon transport coincides with enhanced cold tolerance in leaves, leading to increased TOC content in roots (**Supplementary Figure S5A**). This coordinated response likely explains why roots maintain some level of growth even during the later stages of cold stress.

In addition, plant growth and development are also influenced by the rate of de-adaptation (Vyse et al., 2019). The rate of de-adaptation may depend on the degree of freezing tolerance and plant species (Zuther et al., 2015; Rapacz et al., 2017). Our study revealed that the rate of de-adaptation also differed among organs. The leaf transcriptome responded rapidly to the rising temperature, whereas the root transcriptome responded slowly (**Figure 2C**). Interestingly, we observed that NR and NiR activities remained high in leaves during the recovery stage (**Supplementary Figures S5E, F**). Concurrently, at RD2 and RD7, TN and TOC were preferentially allocated to leaves (**Supplementary Figures S5A, B**). This allocation pattern suggests a growth strategy in *A. annua* during adaptation, where more resources are directed toward leaves, prioritizing the development of the above-ground portion.

## Conclusion

This study employed comprehensive transcriptomic and metabolite quantitative analyses of leaves and roots to investigate cold adaptation and de-adaptation in *A. annua*. Furthermore, it revealed that cold stress facilitated the translocation of root-derived nitrogen in the form of amino acids to leaves, supporting the establishment of cold tolerance mechanisms such as enhanced respiration and synthesis of anti-stress compounds. The study also demonstrated that the distribution of carbon sources in both organs influenced carbon metabolism, including cell wall synthesis and breakdown, as well as the downstream pathway of phenylalanine metabolism. In conclusion, this work provides a novel perspective on plant cold adaptation and de-adaptation by focusing on two critical organs. This approach offers valuable insights for future research on plant responses to abiotic stress.

## Data Availability

The datasets presented in this study can be found in online repositories. The names of the repository/repositories and accession number(s) can be found below: https://www.ncbi.nlm.nih.gov/, PRJNA1089479.

## Supplementary material

Supplementary Figure 1
**(A)** Morphological traits of leaves. **(B)** Morphological traits of roots.

Supplementary Figure 2 Validation of 10 gene expression levels under cold stress using quantitative real-time PCR. **(A, B)** Bars in leaves and roots represent the log2 values of fold changes of the cold stress samples compared to the NH6 (i.e., take the gene expression level of NH6 as 1; three biological replicates). Values are expressed as mean ± SE (n = 3).

Supplementary Figure 3
**(A)** Heatmaps illustrating non-redundant Gene Ontology term enrichment (MF; Molecular Function; FDR < 0.05). The color scale corresponds to the -log10 transformation of the FDR for the enrichment according to Fisher’s exact test. The regulatory trend of the transcripts in each bin (up or down) is indicated. **(B)** Heatmaps illustrating DEGs related to calcium channel and kinase proteins in leaves and roots under cold treatment.

Supplementary Figure 4 Analysis of transcription factors (TFs) in differentially expressed genes (DEGs). **(A)** Proportion of major differential transcription factors in leaves. **(B)** Proportion of major differential transcription factors in roots.

Supplementary Figure 5
**(A, B)** Dynamics of total organic carbon (TOC; **A**) and total nitrogen (TN; **B**) of leaves and roots throughout the experiment. **(C, D)** Nitrate nitrogen (NO3-; **C**) and ammonium nitrogen content (NH4+; **D**). **(E, F)** Nitrate reductase (NR; **E**) and nitrite reductase (NiR; **F**) activity. The abbreviations of DW and FW represent dry weight and fresh weight, respectively.

Supplementary Figure 6 Co-expression analysis of carbohydrate metabolism and amino acid metabolism in leaves and roots under cold treatment.

Supplementary Figure 7
**(A, B)** Comparison of the expression of PSII and PSI genes, LHCI and LHCII genes annotated by the KEGG database under cold stress. **(C)** Boxes showing the expression of ELIPs and FTSH compared with NH6 under cold stress. Boxes represent the log2 values of fold changes of the cold stress samples compared to the NH6. **(D)** Anthocyanin content in leaves. Asterisks indicate significant differences between treatment groups and the control group (NH6). Significance levels are: *P < 0.05; **P < 0.01.

Supplementary Figure 8
**(A)** Changes in the expression of DEGs involved in Auxin, GA, SA, Ethylene, BR and CK biosynthesis in leaves and roots under cold treatment. **(B)** The picture representing changes in various hormone synthesis and signaling pathways. **(C–E)** Changes in the expression of DEGs in Auxin, GA, SA, Ethylene, BR and CK signal pathways in leaves and roots under cold treatment. CK, Cytokinin; ABA, Abscisic acid; BR, Brassinosteroids; GA, Gibberellic acid; ETH, Ethylene; JA, jasmonic acid; SA, Salicylic acid.
