# Supplementary material for: Transcriptome dynamics in Artemisia annua provides new insights into cold adaptation and de-adaptation
Source: Front Plant Sci. 2024 Aug 29;15:1412416. doi: 10.3389/fpls.2024.1412416 (PMC11390472; doi:10.3389/fpls.2024.1412416)
Supplement: Supplementary file 1 [file DataSheet1.zip › Supplementary Table/Supplementary Table 1.pdf]

Supplementary Table 1. Overview of the RNA-seq data

Groups	Sample	Clean reads	Mapped Reads	Clean bases	%≥Q30
Leaves	NH6-1	27,484,005	47,860,782 (87.07%)	8,184,703,864	94.24%
	NH6-2	26,169,508	45,496,959 (86.93%)	7,799,006,114	94.19%
	NH6-3	22,919,634	39,656,893 (86.51%)	6,826,206,634	94.28%
	ND7-1	27,281,870	47,589,019 (87.22%)	8,083,308,078	94.17%
	ND7-2	23,284,731	40,173,633 (86.27%)	6,937,819,224	93.84%
	ND7-3	21,699,857	37,739,271 (86.96%)	6,483,502,682	94.20%
	ND14-1	22,261,191	38,679,629 (86.88%)	6,642,410,824	94.33%
	ND14-2	22,265,179	38,548,039 (86.57%)	6,647,296,240	94.15%
	ND14-3	22,219,548	38,484,742 (86.60%)	6,627,887,142	94.05%
	CH6-1	25,096,939	44,112,655 (87.88%)	7,478,632,996	94.37%
	CH6-2	22,170,199	38,638,465 (87.14%)	6,609,469,130	94.47%
	CH6-3	22,593,799	39,283,063 (86.93%)	6,737,607,694	94.32%
	CD2-1	26,607,176	46,386,711 (87.17%)	7,935,297,724	94.24%
	CD2-2	24,073,874	41,897,727 (87.02%)	7,184,554,444	93.54%
	CD2-3	24,448,794	42,918,185 (87.77%)	7,292,858,228	94.39%
	CD7-1	21,582,846	37,751,986 (87.46%)	6,423,218,068	93.93%
	CD7-2	23,097,032	40,605,557 (87.90%)	6,890,133,896	94.42%
	CD7-3	21,701,633	37,861,777 (87.23%)	6,461,972,044	94.20%
	RH6-1	25,246,187	43,914,611 (86.97%)	7,497,155,960	94.14%
	RH6-2	27,145,915	47,114,475 (86.78%)	8,090,309,528	94.34%
	RH6-3	24,267,921	42,137,411 (86.82%)	7,245,611,084	93.88%
	RD2-1	24,023,349	41,451,358 (86.27%)	7,155,697,876	94.02%
	RD2-2	26,725,250	46,597,638 (87.18%)	7,959,686,048	94.30%
	RD2-3	25,843,604	45,130,387 (87.31%)	7,692,854,880	94.24%
	RD7-1	24,897,045	42,995,260 (86.35%)	7,420,010,336	94.32%
	RD7-2	24,469,845	41,955,457 (85.73%)	7,300,201,004	94.16%
	RD7-3	23,453,539	40,351,321 (86.02%)	6,984,172,794	93.85%
Roots	NH6-1	22,875,720	39,177,677 (85.63%)	6,824,778,780	93.95%
	NH6-2	24,678,334	41,647,952 (84.38%)	7,357,179,254	93.99%
	NH6-3	23,682,709	39,711,074 (83.84%)	7,070,068,320	93.64%
	ND7-1	25,877,544	43,043,437 (83.17%)	7,723,932,676	93.86%
	ND7-2	22,812,957	38,392,461 (84.15%)	6,807,673,920	93.85%
	ND7-3	21,925,742	36,370,770 (82.94%)	6,545,261,756	94.21%
	ND14-1	20,863,755	35,494,276 (85.06%)	6,229,139,622	93.92%
	ND14-2	23,777,918	39,884,891 (83.87%)	7,096,700,524	94.05%
	ND14-3	21,608,744	36,764,552 (85.07%)	6,447,575,942	93.87%
	CH6-1	22,338,232	38,174,471 (85.45%)	6,663,258,188	93.94%
	CH6-2	23,102,385	39,124,679 (84.68%)	6,881,804,884	94.39%
	CH6-3	20,777,866	35,340,543 (85.04%)	6,175,727,892	94.22%
	CD2-1	23,851,319	39,635,305 (86.06%)	6,866,134,218	94.18%
	CD2-2	26,122,014	2,747,440 (81.82%)	7,782,703,086	94.05%
	CD2-3	19,980,056	34,652,642 (86.72%)	5,951,941,012	94.39%
	CD7-1	23,851,319	41,162,551 (86.29%)	7,105,772,572	94.25%
	CD7-2	23,504,344	40,539,336 (86.24%)	6,993,536,218	94.04%
	CD7-3	20,927,663	35,966,825 (85.93%)	6,236,996,074	94.45%
	RH6-1	23,762,161	40,345,889 (84.90%)	7,083,294,710	93.94%
	RH6-2	21,715,303	36,568,844 (84.20%)	6,471,131,596	93.76%
	RH6-3	24,567,280	41,255,170 (83.96%)	7,323,542,888	93.88%
	RD2-1	23,530,829	40,075,702 (85.16%)	7,021,647,716	93.97%
	RD2-2	25,389,261	42,082,932 (82.88%)	7,562,427,112	94.23%
	RD2-3	25,192,859	43,114,085 (85.57%)	7,503,050,968	94.12%
	RD7-1	25,260,505	42,684,965 (84.49%)	7,533,468,352	93.92%
	RD7-2	21,624,385	36,071,813 (83.41%)	6,448,406,900	94.30%
	RD7-3	20,083,727	34,373,298 (85.57%)	5,978,619,018	94.05%
