# Supplementary material for: Transcriptome dynamics in Artemisia annua provides new insights into cold adaptation and de-adaptation
Source: Front Plant Sci. 2024 Aug 29;15:1412416. doi: 10.3389/fpls.2024.1412416 (PMC11390472; doi:10.3389/fpls.2024.1412416)
Supplement: Supplementary file 1 [file DataSheet1.zip › Supplementary Table/Supplementary Table 2.pdf]

Supplementary Table 2. Identification of DEGs (|Log2|>1; FDR<0.05)

Treatment	Groups	Leaves	Roots
Cold stress	CH6 vs NH6	7534	2920
	CD2 vs NH6	11976	5122
	CD7 vs NH6	5592	5052
Recovery	RH6 vs ND7	596	1620
	RD2 vs ND7	2070	1459
	RD7 vs ND14	406	712
