# Supplementary material for: Transcriptome dynamics in Artemisia annua provides new insights into cold adaptation and de-adaptation
Source: Front Plant Sci. 2024 Aug 29;15:1412416. doi: 10.3389/fpls.2024.1412416 (PMC11390472; doi:10.3389/fpls.2024.1412416)
Supplement: Supplementary file 1 [file DataSheet1.zip › Supplementary Table/Supplementary Table 3.pdf]

Supplementary Table 3. DEGs related to transcription factor of leaves and roots

Family	Gene name	Leaf (FPKM)				Annotation	Gene name	Root (FPKM)				Annotation
		NH6	CH6	CD2	CD7			NH6	CH6	CD2	CD7	
AP2/ERF-ERF	Artemisia_annua_newGene_33079	1.54	22.17	0.41	0.23	Ethylene-responsive transcription factor ERF054	CTI12_AA012090	11.63	12.34	24.74	42.90	Dehydration-responsive element-binding protein 3
	Artemisia_annua_newGene_33080	1.10	11.50	0.30	0.32	Ethylene-responsive transcription factor ERF054	CTI12_AA042920	0.85	12.01	15.25	5.06	Pathogenesis-related genes transcriptio--l activator PTI5
	CTI12_AA078540	0.13	6.80	2.20	2.73	Dehydration-responsive element-binding protein 1D	CTI12_AA161760	1.20	3.01	10.66	15.34	Ethylene-responsive transcription factor ERF043
	CTI12_AA212020	3.36	17.52	25.53	5.01	Ethylene-responsive transcription factor ERF010	CTI12_AA061730	0.32	31.54	9.57	3.03	Ethylene-responsive transcription factor 1B
	CTI12_AA238990	2.73	60.86	2.76	5.14	Dehydration-responsive element-binding protein 1E	CTI12_AA548660	1.75	43.91	13.15	5.67	Ethylene-responsive transcription factor 1B
	CTI12_AA257830	18.83	64.47	33.56	20.57	Ethylene-responsive transcription factor ERF118	CTI12_AA019830	53.78	0.54	0.68	0.19	Ethylene-responsive transcription factor ERF023
	CTI12_AA268220	0.14	27.46	1.16	2.49	Dehydration-responsive element-binding protein 1D	CTI12_AA111400	24.45	0.26	0.14	0.11	Dehydration-responsive element-binding protein 1B
	CTI12_AA366070	2.58	15.76	5.07	2.15	Ethylene-responsive transcription factor 4	CTI12_AA111410	18.03	1.16	2.80	3.04	Ethylene-responsive transcription factor ERF027
	CTI12_AA247780	1.36	1.16	68.75	2.68	Pathogenesis-related genes transcriptio--l activator PTI5	CTI12_AA276500	17.88	0.87	0.91	0.69	Ethylene-responsive transcription factor ERF017
	CTI12_AA053710	1.86	19.00	2.42	1.88	Ethylene-responsive transcription factor ERF061	CTI12_AA307510	17.28	0.68	0.34	0.71	Ethylene-responsive transcription factor ERF025
	CTI12_AA101230	14.40	42.70	31.00	38.80	Ethylene-responsive transcription factor WIN1	CTI12_AA330550	34.88	1.29	0.67	1.47	Dehydration-responsive element-binding protein 1D
	CTI12_AA276500	17.26	4.04	0.99	1.18	Ethylene-responsive transcription factor ERF017	CTI12_AA557900	21.77	1.56	3.37	10.25	Dehydration-responsive element-binding protein 3
	CTI12_AA587010	17.35	1.94	0.85	3.15	Ethylene-responsive transcription factor-like protein	CTI12_AA076160	142.89	20.98	61.40	33.52	Ethylene-responsive transcription factor RAP2-4
	CTI12_AA095040	189.67	55.14	44.20	105.11	Ethylene-responsive transcription factor 13						
WRKY	Artemisia_annua_newGene_10883	0.86	7.59	4.70	1.58	Probable WRKY transcription factor 40	CTI12_AA014850	26.38	100.79	68.54	18.83	Probable WRKY transcription factor 54
	Artemisia_annua_newGene_4192	3.07	13.60	21.44	7.71	Probable WRKY transcription factor 26	CTI12_AA054210	14.47	68.69	32.46	15.13	Probable WRKY transcription factor 75
	Artemisia_annua_newGene_90842	1.23	2.95	14.07	2.96	Probable WRKY transcription factor 50	CTI12_AA358100	14.19	41.43	17.64	6.17	WRKY transcription factor WRKY24
	CTI12_AA115020	6.94	34.44	40.26	22.07	WRKY transcription factor 6	CTI12_AA425160	0.55	2.28	6.94	0.67	Probable WRKY transcription factor 70
	CTI12_AA014850	14.68	37.41	114.81	28.35	Probable WRKY transcription factor 54	CTI12_AA456940	1.01	5.24	11.33	3.44	Probable WRKY transcription factor 70
	CTI12_AA054210	5.48	18.19	36.20	6.88	Probable WRKY transcription factor 75	CTI12_AA587740	4.96	17.99	27.58	8.84	Probable WRKY transcription factor 70
	CTI12_AA163270	2.33	13.01	22.48	2.80	Probable WRKY transcription factor 75	CTI12_AA125080	49.34	14.64	6.62	4.49	Probable WRKY transcription factor 41
	CTI12_AA234940	19.33	82.47	110.95	30.10	Probable WRKY transcription factor 33	CTI12_AA162450	41.02	11.90	12.02	14.71	Probable WRKY transcription factor 23
	CTI12_AA259860	24.19	169.17	103.43	41.65	Probable WRKY transcription factor 40	CTI12_AA234940	47.59	83.99	30.52	12.39	Probable WRKY transcription factor 33
	CTI12_AA329040	16.23	126.45	73.90	39.19	Probable WRKY transcription factor 40	CTI12_AA367540	86.50	38.97	26.88	10.58	Probable WRKY transcription factor 46
	CTI12_AA358100	24.93	86.51	62.07	35.31	WRKY transcription factor WRKY24	CTI12_AA449960	39.12	5.19	2.92	1.66	Probable WRKY transcription factor 41
	CTI12_AA364180	0.69	7.12	16.24	1.36	Probable WRKY transcription factor 75	CTI12_AA066530	5.34	0.45	0.74	0.51	Probable WRKY transcription factor 27
	CTI12_AA408680	12.31	58.57	142.64	34.46	Probable WRKY transcription factor 51						
	CTI12_AA449040	4.34	26.28	17.39	1.46	Probable WRKY transcription factor 40						
	CTI12_AA505740	1.46	10.46	9.46	6.45	Probable WRKY transcription factor 23						
	CTI12_AA074230	3.21	29.00	10.05	0.53	Probable WRKY transcription factor 40						
	CTI12_AA014560	5.94	20.25	19.01	9.77	Probable WRKY transcription factor 11						
	CTI12_AA449960	20.03	10.01	3.80	0.75	Probable WRKY transcription factor 41						
MYB	CTI12_AA183300	2.78	28.53	6.08	3.97	Transcription factor MYB12	Artemisia_annua_newGene_79983	2.23	18.84	3.34	0.72	Transcription factor MYB14
	CTI12_AA326620	3.88	2.26	18.28	4.84	Transcription factor MYB62	CTI12_AA208460	1.89	18.53	15.54	3.19	Transcription factor MYB78
	CTI12_AA472470	10.84	56.46	131.64	18.07	Transcription factor MYB13	CTI12_AA252800	2.51	18.97	2.37	0.75	Transcription factor MYB14
	CTI12_AA281860	6.67	3.39	0.97	1.11	Transcription factor MYB16	CTI12_AA420020	48.85	157.20	49.69	24.63	Transcription factor MYB15
	CTI12_AA370020	12.12	5.56	7.62	2.24	Transcription factor MYB73	CTI12_AA125430	5.05	1.38	0.60	0.78	Transcription factor MYB44
	CTI12_AA068790	50.38	15.14	2.42	0.79	Transcription factor MYB111	CTI12_AA280420	18.41	0.97	1.71	5.14	Transcription factor MYB73
	CTI12_AA608220	24.95	13.50	1.36	0.43	Transcription factor MYB111	CTI12_AA335470	40.23	9.05	13.41	28.74	Transcription factor MYB61
							CTI12_AA389460	12.03	5.87	1.75	1.05	Transcription factor MYB14
							CTI12_AA370020	51.46	2.49	9.19	10.31	Transcription factor MYB73
							CTI12_AA572640	44.55	3.33	6.17	11.94	Transcription factor MYB73
							CTI12_AA068790	9.43	0.48	0.97	0.32	Transcription factor MYB111
							CTI12_AA107580	9.64	2.82	2.07	1.01	Transcription factor MYB2
NAC	Artemisia_annua_newGene_79513	0.01	1.79	8.13	1.27	NAC domain-containing protein 90	CTI12_AA120720	8.06	37.09	41.51	27.32	NAC domain-containing protein 72
	Artemisia_annua_newGene_96620	5.29	19.97	13.77	3.91	NAC domain-containing protein 14	CTI12_AA145460	2.47	9.63	15.11	7.89	NAC domain-containing protein 72
	CTI12_AA120720	12.39	134.06	174.01	77.82	NAC domain-containing protein 72	CTI12_AA122010	29.19	76.22	85.34	65.82	NAC domain-containing protein 2
	CTI12_AA136530	7.21	79.43	22.35	10.62	NAC domain-containing protein 83	CTI12_AA044270	8.04	4.20	3.97	1.58	NAC domain-containing protein 73
	CTI12_AA145460	5.23	52.80	38.93	33.83	NAC domain-containing protein 72						
	CTI12_AA122010	44.54	338.79	246.63	157.72	NAC domain-containing protein 2						
	CTI12_AA184530	30.76	153.92	163.62	140.97	NAC domain-containing protein 83						
	CTI12_AA231670	2.48	12.16	5.96	4.64	NAC domain containing protein 50						
	CTI12_AA238640	9.72	41.34	51.46	14.11	NAC domain-containing protein 83						
	CTI12_AA255460	1.26	20.86	10.41	14.23	NAC domain-containing protein 100						
	CTI12_AA299090	5.08	32.29	43.25	10.19	NAC domain containing protein 50						
	CTI12_AA354210	32.39	135.95	80.63	54.66	NAC domain-containing protein 91						
	CTI12_AA396830	0.72	16.95	1.75	1.10	NAC domain-containing protein 68						
	CTI12_AA466340	1.49	26.22	35.62	46.15	NAC domain-containing protein 21/22						
	CTI12_AA494790	0.00	12.96	30.11	84.65	NAC transcription factor 29						
	CTI12_AA525490	0.53	6.06	9.79	1.25	NAC domain-containing protein 90						
	CTI12_AA559880	0.93	13.73	1.82	0.77	NAC domain-containing protein 68						
	CTI12_AA609230	2.20	21.06	16.11	15.31	NAC domain-containing protein 100						
	CTI12_AA108260	28.50	341.18	109.32	28.71	NAC domain-containing protein 2						
bHLH	CTI12_AA166270	1.685	9.176	3.577	8.710	Transcription factor PIF7	CTI12_AA246520	0.900	4.042	0.250	0.852	Transcription factor bHLH66
	CTI12_AA207600	1.279	1.541	4.720	6.078	Transcription factor bHLH25	CTI12_AA397770	6.558	2.124	0.927	1.449	Transcription factor bHLH25
	CTI12_AA006590	10.887	36.668	5.135	1.311	Transcription factor bHLH35	CTI12_AA445980	17.306	3.232	1.145	3.178	Transcription factor ABA-INDUCIBLE bHLH-TYPE
	CTI12_AA253760	0.445	3.407	5.505	2.621	Transcription factor bHLH106	CTI12_AA459270	42.936	14.922	12.621	19.482	Transcription factor bHLH62
	CTI12_AA344800	2.962	20.383	19.501	22.751	Transcription factor BEE 1	CTI12_AA061480	10.108	14.738	0.333	1.282	Transcription factor bHLH93
	CTI12_AA372230	4.754	9.633	36.886	10.884	Transcription factor bHLH62	CTI12_AA544930	5.193	0.474	0.547	0.620	Transcription factor bHLH25
	CTI12_AA396330	6.824	58.602	4.517	1.966	Transcription factor bHLH35	CTI12_AA601930	21.805	6.601	3.000	3.704	Transcription factor bHLH130
	CTI12_AA601930	3.049	23.937	7.363	11.859	Transcription factor bHLH130						
	CTI12_AA617370	0.990	4.975	0.533	0.462	Transcription factor bHLH35						
	CTI12_AA077530	3.549	12.588	24.918	11.845	Transcription factor bHLH106						
	Artemisia_annua_newGene_92045	7.263	1.689	0.764	2.591	Transcription factor BHLH094						
	CTI12_AA115860	7.096	1.472	0.393	0.482	Transcription factor bHLH62						
	CTI12_AA090870	37.170	48.560	9.760	18.541	Transcription factor PIF1						
	CTI12_AA028320	8.031	1.867	3.191	4.105	Transcription factor bHLH62						
	CTI12_AA323900	9.992	2.442	1.972	5.183	Transcription factor bHLH63						
	CTI12_AA349550	9.082	1.589	1.714	2.311	Transcription factor bHLH14						
	CTI12_AA351610	24.333	4.821	5.260	17.155	Transcription factor bHLH63						
	CTI12_AA445980	12.749	13.230	0.995	1.157	Transcription factor ABA-INDUCIBLE bHLH-TYPE						
	CTI12_AA491840	28.682	7.049	5.383	7.092	Transcription factor bHLH62						
	CTI12_AA603780	5.759	2.041	0.903	1.994	Transcription factor ICE1						
bZIP	CTI12_AA339020	8.75	63.46	42.44	19.23	Common plant regulatory factor 1	CTI12_AA246520	0.90	4.04	0.25	0.85	Transcription factor bHLH66
	CTI12_AA345640	8.92	167.95	47.00	47.11	bZIP transcription factor 12	CTI12_AA006590	18.92	6.24	9.63	2.71	Transcription factor bHLH35
	CTI12_AA491760	4.84	104.39	15.96	7.09	Transcription factor HY5	CTI12_AA367290	3.64	0.03	1.05	2.62	Transcription factor bHLH118
	CTI12_AA059690	2.05	6.98	15.93	6.91	bZIP transcription factor 44	CTI12_AA397770	6.56	2.12	0.93	1.45	Transcription factor bHLH25
	CTI12_AA042360	60.74	132.58	55.50	45.09	ABSCISIC ACID-INSENSITIVE 5-like protein 7	CTI12_AA445980	17.31	3.23	1.15	3.18	Transcription factor ABA-INDUCIBLE bHLH-TYPE
	CTI12_AA604700	3.54	125.41	12.45	5.18	Transcription factor HY5	CTI12_AA459270	42.94	14.92	12.62	19.48	Transcription factor bHLH62
	CTI12_AA098120	4.15	30.56	23.55	7.95	Common plant regulatory factor 1	CTI12_AA491840	31.63	8.20	16.82	18.77	Transcription factor bHLH62
	CTI12_AA295800	9.34	0.88	0.46	2.24	Basic leucine zipper 34	CTI12_AA061480	10.11	14.74	0.33	1.28	Transcription factor bHLH93
							CTI12_AA544930	5.19	0.47	0.55	0.62	Transcription factor bHLH25
							CTI12_AA596120	3.49	0.96	0.35	0.77	Transcription factor bHLH14
							CTI12_AA604690	3.76	0.67	1.11	0.60	Transcription factor bHLH14
