# Supplementary material for: Transcriptome dynamics in Artemisia annua provides new insights into cold adaptation and de-adaptation
Source: Front Plant Sci. 2024 Aug 29;15:1412416. doi: 10.3389/fpls.2024.1412416 (PMC11390472; doi:10.3389/fpls.2024.1412416)
Supplement: Supplementary file 1 [file DataSheet1.zip › Supplementary Table/Supplementary Table 4.pdf]

Supplementary Table 4. DEGs related to linoleic acid metabolism, alpha-linolenic acid metabolism and Biosynthesis of unsaturated fatty acids

Groups	Gene name	Groups				Annotation
		NH6	CH6	CD2	CD7	
Leaves	CTI12_AA049700	35.55069033	164.506898	50.18309433	47.56767133	Artemisinic aldehyde Delta(11(13)) reductase
	CTI12_AA049710	11.548582	9.332353333	2.630577667	5.359703667	Artemisinic aldehyde Delta(11(13)) reductase
	Artemisia_annua_newGene_53186	0.341391	3.096689333	0.374114333	0.159712667	12-oxophytodienoate reductase 2
	CTI12_AA141940	6.387667	3.715853667	1.175242333	1.587362	12-oxophytodienoate reductase 2
	CTI12_AA450280	3.092661667	11.482079	7.350654333	5.073034333	Putative 12-oxophytodienoate reductase 11
	CTI12_AA116560	54.120974	129.1104637	159.555903	93.480077	3-ketoacyl-CoA thiolase 2
	CTI12_AA584370	23.47311667	51.12388567	24.14333667	8.861390667	3-ketoacyl-CoA thiolase 2
	CTI12_AA419030	14.84036033	21.28922167	36.869649	16.86475633	4-coumaratenaCoA ligase-like 5
	CTI12_AA094450	12.01518033	8.775244667	19.86827367	22.33884267	Acyl-coenzyme A oxidase 2
	CTI12_AA409890	42.09972933	37.89798067	92.761173	62.38397567	Acyl-coenzyme A oxidase 3
	CTI12_AA188500	5.871154667	1.239691333	0.641797667	0.329683333	Acyl-coenzyme A oxidase 4
	CTI12_AA497390	1.063426667	10.355382	17.41300933	7.786580667	Alcohol dehydrogenase 1
	CTI12_AA305120	40.24730433	202.2301127	58.64888633	16.47436833	Allene oxide cyclase
	CTI12_AA606960	23.224632	97.92578767	34.407919	12.63962833	Allene oxide cyclase
	CTI12_AA160350	0	0.995653	7.379234333	0.372737333	Allene oxide synthase
	CTI12_AA274240	25.56307033	51.28072967	32.16834733	32.47165367	Allene oxide synthase
	Artemisia_annua_newGene_84146	8.275216333	26.775571	110.909076	20.39786333	Alpha-dioxygenase 1
	CTI12_AA382980	1.996841667	0.433723667	0.282622	0.448438667	Alpha-dioxygenase 1
	CTI12_AA631020	0.808027667	3.429349667	14.40888	3.420023	Alpha-dioxygenase 1
	CTI12_AA468230	137.778468	78.63999067	43.738641	16.78711033	Fatty acid hydroperoxide lyase
	CTI12_AA608020	4.884392667	2.492024	2.820559333	0.046787333	Fatty acid hydroperoxide lyase
	Artemisia_annua_newGene_81328	1.506210667	1.935583	0.087310.341842333		Jasmonate O-methyltransferase
	CTI12_AA259560	5.349699667	5.753835667	0.260408333	1.356347667	Jasmonate O-methyltransferase
	Artemisia_annua_newGene_74946	0.850115333	0.218944	0.057986667	0.594389333	Linoleate 13S-lipoxygenase 2-1
	Artemisia_annua_newGene_85102	2.22888	1.816132667	0	0.087511667	Linoleate 13S-lipoxygenase 2-1
	Artemisia_annua_newGene_9080	2.689248667	0.413369667	0.024379333	0.264945667	Linoleate 13S-lipoxygenase 2-1
	CTI12_AA192480	234.08125	171.0889173	46.702238	12.080033	Linoleate 13S-lipoxygenase 2-1
	CTI12_AA222610	135.214709	93.494448	12.09480433	18.92971	Linoleate 13S-lipoxygenase 2-1
	CTI12_AA238950	40.48780833	24.93512833	13.545223	9.584877333	Linoleate 13S-lipoxygenase 2-1
	CTI12_AA269640	83.394488	43.64836133	4.440569333	8.016031667	Linoleate 13S-lipoxygenase 2-1
	CTI12_AA126410	9.384797333	6.825025	1.984058667	1.958057	Linoleate 13S-lipoxygenase 2-1
	CTI12_AA442740	54.24171	24.867319	3.506760667	8.391427	Linoleate 13S-lipoxygenase 2-1
	CTI12_AA452180	119.5101103	69.02291	11.400959	34.16434733	Linoleate 13S-lipoxygenase 2-1
	CTI12_AA206500	28.74552967	18.54240567	15.615068	12.31637267	Linoleate 13S-lipoxygenase 3-1
	CTI12_AA082020	21.314115	40.67199833	24.028196	6.910964	Linoleate 13S-lipoxygenase 3-1
	CTI12_AA008440	16.558776	14.57233267	11.25790333	7.002138333	Linoleate 13S-lipoxygenase 3-1
	CTI12_AA096310	1.033424667	0.247833333	0.052719	0.312753667	Probable lipoxygenase 6
	CTI12_AA433900	132.2165367	26.581679	2.038393667	8.115430667	linoleate 13S-lipoxygenase 2-1,
	CTI12_AA139370	44.728893	81.36620333	111.2966813	86.032749	Peroxisomal acyl-coenzyme A oxidase 1 OS
	CTI12_AA262600	4.397942667	5.065504	3.856133	1.038962667	Peroxisomal acyl-coenzyme A oxidase 1
	CTI12_AA010440	9.215912667	6.62981	1.169483333	2.496129667	Peroxisomal acyl-coenzyme A oxidase 1
	Artemisia_annua_newGene_42868	5.153914	1.596156333	1.636335667	3.28922	Peroxisomal fatty acid beta-oxidation multifunctional protein AIM1
	CTI12_AA221610	21.43594433	6.587244667	3.727446667	7.234300333	Peroxisomal fatty acid beta-oxidation multifunctional protein AIM1
	CTI12_AA241820	11.37028267	3.846406333	2.408287667	6.337460667	Peroxisomal fatty acid beta-oxidation multifunctional protein AIM1
	CTI12_AA592830	15.25935233	2.837590333	1.484610333	2.75784	Peroxisomal fatty acid beta-oxidation multifunctional protein AIM1
	Artemisia_annua_newGene_51692	14.572265	15.77580933	4.506492333	0.385155667	Salicylate carboxymethyltransferase
	CTI12_AA273750	1.842296	3.705712	0.829159	0.400104	Salicylate carboxymethyltransferase
	CTI12_AA130660	12.099062	16.901282	4.652287	0.577405333	Salicylate carboxymethyltransferase
	CTI12_AA520710	11.116307	10.265404	32.11218	33.60012	Triacylglycerol lipase SDP1
	CTI12_AA603150	4.828049333	1.838452667	4.534929	6.875778	Triacylglycerol lipase SDP1
	CTI12_AA116560	54.120974	129.1104637	159.555903	93.480077	3-ketoacyl-CoA thiolase 2
	CTI12_AA584370	23.47311667	51.12388567	24.14333667	8.861390667	3-ketoacyl-CoA thiolase 2
	Artemisia_annua_newGene_47908	0	7.124300667	13.51345033	1.088476667	Delta(12) fatty acid desaturase DES8.11
	Artemisia_annua_newGene_47909	0	1.031996667	10.21284867	0.225128	Delta(12) fatty acid desaturase DES8.11
	Artemisia_annua_newGene_5917	5.665134667	14.91651533	21.973552	4.904059333	Delta(12) fatty acid desaturase DES8.11
	Artemisia_annua_newGene_81933	0.843773667	3.610813	5.080328667	1.541744333	Delta(12) fatty acid desaturase DES8.11
	Artemisia_annua_newGene_81934	1.743952	5.34479	7.938607	1.752689	Delta(12) fatty acid desaturase DES8.11
	CTI12_AA160960	27.614536	66.00927733	180.0020447	41.07231433	Delta(12) fatty acid desaturase DES8.11
	CTI12_AA185670	0.547763333	4.102758333	28.199417	3.952189	Delta(12) fatty acid desaturase DES8.11
	CTI12_AA195950	42.638910	8.746066	3.169135	4.708906667	Delta(12) fatty acid desaturase DES8.11
	CTI12_AA224170	1.805969333	13.34524167	43.40623467	5.618091	Delta(12) fatty acid desaturase DES8.11
	CTI12_AA282360	0.928984333	2.493810667	38.91961067	6.093833333	Delta(12) fatty acid desaturase DES8.11
	CTI12_AA319560	1.447705	11.173072	54.29242633	9.613793	Delta(12) fatty acid desaturase DES8.11
	CTI12_AA331080	1.602922333	24.166444	37.30654267	3.119672667	Delta(12) fatty acid desaturase DES8.11
	CTI12_AA380280	1.988948333	8.626733333	57.094995	14.964748	Delta(12) fatty acid desaturase DES8.11
	CTI12_AA478830	4.878835333	20.58124933	24.80635867	7.254938	Delta(12) fatty acid desaturase DES8.11
	CTI12_AA569880	1.093040333	4.307815667	21.43479967	3.934153333	Delta(12) fatty acid desaturase DES8.11
	CTI12_AA048090	1.1494	1.701569667	3.989132333	1.146946333	Delta(12) fatty acid desaturase DES8.11
	CTI12_AA589690	3.126771	8.278780333	73.72074833	5.65666	Delta(12) fatty acid desaturase DES8.11
	CTI12_AA595500	0.130607667	0.503715667	4.630297333	0.015250333	Delta(12) fatty acid desaturase DES8.11
	CTI12_AA124210	51.04685333	160.9408773	266.435079	96.14720633	Delta(12) fatty acid desaturase FAD2
	CTI12_AA223670	109.8001507	128.2758307	53.282336	50.72697433	Delta(12) fatty acid desaturase FAD2
	CTI12_AA124190	19.18854933	55.72321933	139.13165	28.529757	Delta(12)-acyl-lipid-desaturase
	CTI12_AA196910	7.189955667	30.29725733	31.41037	9.091130333	Delta(12)-acyl-lipid-desaturase
	CTI12_AA205810	4.460030333	17.47461467	64.17564133	13.58739833	Delta(12)-acyl-lipid-desaturase
	CTI12_AA158090	0.278996	3.325610667	3.917094333	0.376065	Delta(12)-acyl-lipid-desaturase
	CTI12_AA275800	9.306413	7.37457467	130.2260717	24.38608733	Delta(12)-acyl-lipid-desaturase
	CTI12_AA384120	0.852139	10.95426433	12.96162267	0.874254	Delta(12)-acyl-lipid-desaturase
	CTI12_AA525170	4.062691	26.57522433	129.594091	17.24303533	Delta(12)-acyl-lipid-desaturase
	CTI12_AA584700	1.344254	12.73977733	17.797395	1.627211667	Delta(12)-acyl-lipid-desaturase
	CTI12_AA139370	44.728893	81.36620333	111.2966813	86.032749	Peroxisomal acyl-coenzyme A oxidase 1
	CTI12_AA262600	4.397942667	5.065504	3.856133	1.038962667	Peroxisomal acyl-coenzyme A oxidase 1
	CTI12_AA010440	9.215912667	6.62981	1.169483333	2.496129667	Peroxisomal acyl-coenzyme A oxidase 1
	CTI12_AA409890	42.09972933	37.89798067	92.761173	62.38397567	Acyl-coenzyme A oxidase 3
	CTI12_AA188500	5.871154667	1.239691333	0.641797667	0.329683333	Acyl-coenzyme A oxidase 4
	Artemisia_annua_newGene_54779	5.700235667	8.675414667	17.50297133	10.141877	Stearoyl-[acyl-carrier-protein] 9-desaturase
	CTI12_AA518110	3.531979667	4.383898	6.741477333	8.436126667	Very-long-chain (3R)-3-hydroxyacyl-CoA dehydratase PASTICCINO 2B
Roots	CTI12_AA377010	10.96391867	37.85663533	25.109316	48.70660567	12-oxophytodienoate reductase 1
	CTI12_AA406910	6.487565333	2.396401	5.482992.449628333		12-oxophytodienoate reductase 1
	CTI12_AA141940	11.42200267	12.45325.150007333		6.482319667	12-oxophytodienoate reductase 2
	CTI12_AA450280	2.700405667	10.51032367	4.705625667	6.766022	Putative 12-oxophytodienoate reductase 11
	CTI12_AA584370	89.30954133	46.28202633	35.27666267	20.03756367	3-ketoacyl-CoA thiolase 2
	Artemisia_annua_newGene_2180	42.325652	51.933033	11.25467433	17.87200367	Alcohol dehydrogenase 1
	CTI12_AA361200	61.576354	50.24927933	11.410536	31.91607433	Alcohol dehydrogenase 1
	CTI12_AA497390	4.372177333	47.45442733	94.17102367	95.69789733	Alcohol dehydrogenase 1
	Artemisia_annua_newGene_97858	16.40953567	2.748553333	0.722317	3.616616333	Alcohol dehydrogenase 3
	CTI12_AA606960	63.116772	30.19487033	25.86793067	18.40522833	Allene oxide cyclase
	CTI12_AA411880	26.99522233	34.757904	12.471802	11.90517233	Allene oxide synthase 3
	CTI12_AA248020	5.404922667	15.82099467	16.220785	9.906051333	Alpha-dioxygenase 1
	CTI12_AA032350	0.087262	0.646712	0.277767667	1.042175667	Alpha-dioxygenase 1
	CTI12_AA550130	0.205726667	1.200920667	0.768892333	2.015543	Alpha-dioxygenase 1
	CTI12_AA262600	1.291227333	0.224542	5.486368667	0.044167667	Peroxisomal acyl-coenzyme A oxidase 1
	CTI12_AA188500	8.239943667	1.298630667	1.959165	1.533035	Acyl-coenzyme A oxidase 4
	CTI12_AA221610	23.47739333	13.509471	8.677740667	12.78595167	Peroxisomal fatty acid beta-oxidation multifunctional protein AIM1
	CTI12_AA241820	14.32080733	8.345518	5.511075333	11.31245533	Peroxisomal fatty acid beta-oxidation multifunctional protein AIM1
	CTI12_AA592830	11.331194	2.758121667	2.708816333	4.383973667	Peroxisomal fatty acid beta-oxidation multifunctional protein AIM1
	Artemisia_annua_newGene_51692	32.68465233	20.63262567	58.50538033	4.251762	Salicylate carboxymethyltransferase
	CTI12_AA130660	22.98706667	18.36807933	48.849111	5.136278333	Salicylate carboxymethyltransferase
	CTI12_AA584370	89.30954133	46.28202633	35.27666267	20.03756367	3-ketoacyl-CoA thiolase 2
	CTI12_AA188500	8.239943667	1.298630667	1.959165	1.533035	Acyl-coenzyme A oxidase 4
	CTI12_AA160960	53.29162867	64.71397767	109.3644587	21.29045867	Delta(12) fatty acid desaturase DES8.11
	CTI12_AA185670	0.382360667	11.74244767	2.778876667	1.54911	Delta(12) fatty acid desaturase DES8.11
	CTI12_AA195950	66.79870167	1.638053667	48.815621	28.82045433	Delta(12) fatty acid desaturase DES8.11
	CTI12_AA224170	3.938213333	17.043114	17.26820967	3.371318333	Delta(12) fatty acid desaturase DES8.11
	CTI12_AA319560	1.811339333	13.75502067	12.38295933	2.554438	Delta(12) fatty acid desaturase DES8.11
	CTI12_AA331080	1.9452229.07258833		24.98848067	9.647004	Delta(12) fatty acid desaturase DES8.11
	CTI12_AA389900	10.38871633	4.866914667	4.001807	2.086691	Delta(12) fatty acid desaturase DES8.11
	CTI12_AA380280	1.241713333	41.47116367	16.297352	4.518942	Delta(12) fatty acid desaturase DES8.11
	CTI12_AA424080	1.653098	0	0.006862667	1.365016	Delta(12) fatty acid desaturase DES8.11
	CTI12_AA478830	27.54048867	63.07787367	50.28481967	23.66922733	Delta(12) fatty acid desaturase DES8.11
	CTI12_AA048090	4.877915667	15.19636533	23.08589933	6.929237333	Delta(12) fatty acid desaturase DES8.11
	CTI12_AA589690	7.023329	8.601461333	13.561267	3.150533667	Delta(12) fatty acid desaturase DES8.11
	CTI12_AA526780	22.44390633	0	71.76760833	10.83523933	Delta(12) fatty acid desaturase FAD2
	CTI12_AA205810	6.192271	35.12965267	31.96004167	2.539003	Delta(12)-acyl-lipid-desaturase
	CTI12_AA275800	19.75634433	33.95507167	78.59761367	17.02442067	Delta(12)-acyl-lipid-desaturase
	CTI12_AA384120	2.842819	6.237462333	3.904551.03712		Delta(12)-acyl-lipid-desaturase
	CTI12_AA525170	4.064727333	24.49594867	18.084825	4.457578667	Delta(12)-acyl-lipid-desaturase
	CTI12_AA584700	4.264345667	14.149354	7.305209333	3.577412	Delta(12)-acyl-lipid-desaturase
	CTI12_AA006820	3.220594667	1.263127	0.658333	0.255935	NADPH-dependent aldehyde reductase-like protein
	CTI12_AA262600	1.291227333	0.224542	5.486368667	0.044167667	Peroxisomal acyl-coenzyme A oxidase 1
	CTI12_AA109640	13.22045533	13.088353	0.907394667	0.632621333	Stearoyl-[acyl-carrier-protein] 9-desaturase 6
	Artemisia_annua_newGene_54778	24.03771567	34.50276133	54.68910767	36.93263233	Stearoyl-[acyl-carrier-protein] 9-desaturase
	Artemisia_annua_newGene_54779	2.017734	6.441045	6.500112333	6.658081667	Stearoyl-[acyl-carrier-protein] 9-desaturase
	Artemisia_annua_newGene_65056	1.300455667	7.209563	4.309226	9.504172	Stearoyl-[acyl-carrier-protein] 9-desaturase
	CTI12_AA414770	18.045702	18.53215467	28.19117	48.98993933	Very-long-chain 3-oxoacyl-CoA reductase 1
	CTI12_AA512330	1.371227333	1.271171667	1.461705333	4.198587667	Very-long-chain enoyl-CoA reductase
