# Supplementary material for: Transcriptome dynamics in Artemisia annua provides new insights into cold adaptation and de-adaptation
Source: Front Plant Sci. 2024 Aug 29;15:1412416. doi: 10.3389/fpls.2024.1412416 (PMC11390472; doi:10.3389/fpls.2024.1412416)
Supplement: Supplementary file 1 [file DataSheet1.zip › Supplementary Table/Supplementary Table 5.pdf]

Supplementary Table S5. DEGs annotations were MATe Family ,ABCG transporter and Dirigent-like protein

Family	Gene name	Groups				Annotation
		NH6	CH6	CD2	CD7	
MATE family	Artemisia_annua_newGene_23564	13.07566067	25.89945533	52.11435733	27.81904133	Protein DETOXIFICATION 38
MATE family	Artemisia_annua_newGene_23932	18.54250867	53.60643133	59.62836267	44.284032	Protein DETOXIFICATION 24
MATE family	Artemisia_annua_newGene_59218	0.698887	2.946002667	2.774973333	4.534902333	Protein DETOXIFICATION 16
MATE family	Artemisia_annua_newGene_59345	5.764991333	16.48490967	75.83874767	85.11841067	Protein DETOXIFICATION 42
MATE family	Artemisia_annua_newGene_97984	1.762429	4.853909667	24.92717567	10.09341733	Protein DETOXIFICATION 16
MATE family	CTI12_AA116680	3.611289667	7.635318	30.13856467	34.874503	Protein DETOXIFICATION 27
MATE family	CTI12_AA139920	5.668105333	13.11400367	17.78432167	13.592211	Protein DETOXIFICATION 19
MATE family	CTI12_AA144030	3.283400667	15.54450867	54.34987367	43.516049	Protein DETOXIFICATION 27
MATE family	CTI12_AA155620	1.209272667	0.875186667	2.824466333	1.954242333	Protein DETOXIFICATION 14
MATE family	CTI12_AA175410	2.816024333	15.02578467	50.917874	40.81642933	Protein DETOXIFICATION 27
MATE family	CTI12_AA097430	1.820889333	3.383148	4.192682	2.434424	Protein DETOXIFICATION 46
MATE family	CTI12_AA189600	2.948471667	2.519026667	7.910040667	8.389499	Protein DETOXIFICATION 45
MATE family	CTI12_AA196330	30.39022333	39.12021	129.2493403	55.58290167	Protein DETOXIFICATION 40
MATE family	CTI12_AA222400	12.88551133	40.22887233	50.14463233	55.626365	Protein DETOXIFICATION 44
MATE family	CTI12_AA245620	52.02881033	149.1381817	180.3066423	95.76866267	Protein DETOXIFICATION 31
MATE family	CTI12_AA262090	1.52114	10.30562167	6.722002	1.560153667	Protein DETOXIFICATION 49
MATE family	CTI12_AA042560	0.823306667	7.455032333	29.385978	15.66558133	Protein DETOXIFICATION 16
MATE family	CTI12_AA462480	2.818923667	3.324617333	6.981388667	8.912045	Protein DETOXIFICATION 45
MATE family	CTI12_AA525710	2.94858	13.002601	16.216828	13.00509733	Protein DETOXIFICATION 16
MATE family	CTI12_AA065220	2.778408333	8.709624	41.51171967	35.58530567	Protein DETOXIFICATION 12
MATE family	CTI12_AA558780	1.170361667	5.007062333	13.162493	31.62161967	Protein DETOXIFICATION 42
MATE family	CTI12_AA594470	10.57288633	30.75327433	184.9360153	67.48687167	Protein DETOXIFICATION 16
MATE family	CTI12_AA615100	1.906998333	6.501668667	24.24751867	23.247094	Protein DETOXIFICATION 27
MATE family	CTI12_AA261560	2.228759	8.454746667	12.44070167	8.756534	Protein DETOXIFICATION 16
ABC transporter	CTI12_AA225180	1.513120667	3.527800333	8.358034	4.949169	ABC transporter A family member 7
ABC transporter	Artemisia_annua_newGene_10452	2.113299667	7.445692	10.829876	5.912066	ABC transporter B family member 21
ABC transporter	CTI12_AA188770	2.750708	11.66966467	13.69102833	6.908064333	ABC transporter B family member 21
ABC transporter	CTI12_AA268390	2.520903	7.003589667	9.498773667	6.217992667	ABC transporter B family member 21
ABC transporter	CTI12_AA026380	19.864972	35.38358433	38.67358867	40.360606	ABC transporter B family member 25
ABC transporter	CTI12_AA269750	14.531252	25.216576	35.10633967	32.108121	ABC transporter B family member 25
ABC transporter	CTI12_AA413260	14.53440633	26.95185067	46.25432767	52.94413967	ABC transporter B family member 25
ABC transporter	CTI12_AA044490	2.424668333	4.230805	17.90173467	15.08381833	ABC transporter B family member 25
ABC transporter	CTI12_AA464650	5.204888333	8.805405667	28.915721	22.257057	ABC transporter B family member 25
ABC transporter	CTI12_AA290590	15.961167	16.39145967	36.195939	49.69814933	ABC transporter B family member 26
ABC transporter	CTI12_AA326540	8.798522333	6.065589667	5.605346	22.74300533	ABC transporter B family member 28
ABC transporter	CTI12_AA447480	2.669614333	6.641978	9.331052	15.38371567	ABC transporter B family member 28
ABC transporter	CTI12_AA408610	2.691186	26.98009433	67.79250933	33.03301733	ABC transporter C family member 10
ABC transporter	CTI12_AA153640	17.84179133	25.66321733	44.564535	61.534914	ABC transporter C family member 12
ABC transporter	Artemisia_annua_newGene_38516	15.265717	16.50738967	11.44306	34.16899533	ABC transporter C family member 2
ABC transporter	CTI12_AA138490	35.10690767	45.68174333	66.89042233	88.21332467	ABC transporter C family member 2
ABC transporter	CTI12_AA145310	0.302308333	0.262020333	1.938758333	8.966006667	ABC transporter C family member 3
ABC transporter	CTI12_AA414420	5.156609	55.930492	44.145986	18.46831433	ABC transporter C family member 4
ABC transporter	CTI12_AA556750	57.14352267	107.8706137	122.6338283	178.2763977	ABC transporter C family member 4
ABC transporter	CTI12_AA154420	4.530265	13.60137467	15.412883	10.29902533	ABC transporter C family member 8
ABC transporter	CTI12_AA210750	0.238640333	0.263045	0.570647667	1.742430667	ABC transporter G family member 17
ABC transporter	CTI12_AA006320	27.265037	144.2473457	141.0311043	161.944487	ABC transporter G family member 22
ABC transporter	CTI12_AA504770	4.468704333	24.06700433	27.73149933	19.06603067	ABC transporter G family member 22
ABC transporter	CTI12_AA221660	5.581260333	10.528318	24.671764	17.418201	ABC transporter G family member 24
ABC transporter	CTI12_AA018600	0.408684667	0.433477333	0.244776667	1.390516333	ABC transporter G family member 32
	CTI12_AA061840	7.490183333	117.6431883	211.203069	22.96079867	Dirigent protein 23 OS
	CTI12_AA196660	0.143908333	0.924731333	2.583575333	1.575034333	Dirigent protein 23 OS
	CTI12_AA223810	7.065627333	84.531367	122.5952553	16.57591267	Dirigent protein 19 OS
	CTI12_AA238980	31.07470767	119.8126273	87.89726767	26.20006033	Dirigent protein 19 OS
	CTI12_AA306910	5.534275333	58.464775	90.56377933	25.86716033	Dirigent protein 19 OS
	CTI12_AA306930	1.575857333	1.647725333	5.579825333	3.540854	Dirigent protein 19 OS
	CTI12_AA324290	2.914838	15.081841	10.14061367	1.363085667	Dirigent protein 23 OS
Dirigent-like protein	CTI12_AA352670	1.452841	6.488206	5.609844333	1.842658333	Dirigent protein 21 OS
	CTI12_AA357930	0.517861667	7.796787	9.791241	1.571301333	Dirigent protein 19 OS
	CTI12_AA043010	0.129191	0.213918333	2.173655667	0.538233333	Dirigent protein 23 OS
	CTI12_AA365000	4.579248667	13.69750367	17.32709233	4.897361	Dirigent protein 19 OS
	CTI12_AA329080	3.632457333	34.97544167	148.455574	17.97692033	Dirigent protein 23 OS
	CTI12_AA461260	29.07667133	124.8404517	74.856538	34.492091	Dirigent protein 19 OS
	CTI12_AA485940	0.490185667	10.441749	15.94394367	2.300151	Dirigent protein 19 OS
	CTI12_AA372400	8.524890333	53.767341	38.36397433	5.390035333	Dirigent protein 23 OS
	CTI12_AA518470	0.504892667	1.192002333	1.945838	1.035911	Dirigent protein 23 OS
