# Supplementary material for: Transcriptome dynamics in Artemisia annua provides new insights into cold adaptation and de-adaptation
Source: Front Plant Sci. 2024 Aug 29;15:1412416. doi: 10.3389/fpls.2024.1412416 (PMC11390472; doi:10.3389/fpls.2024.1412416)
Supplement: Supplementary file 1 [file DataSheet1.zip › Supplementary Table/Supplementary Table 6.pdf]

Supplementary Table 6. Other modules and GO analysis in WGCNA.

Module	Go terms	FDR	Expression
Grey60	Transcription factor activity	2. 32E- 09	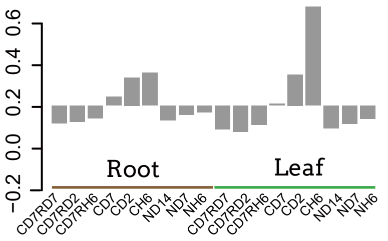
	Amino acid import	2. 07E- 05	
	Sequence-specific DNA binding	4. 72E- 06	
	Abscisic acid-activated signaling pathway	0. 0108	
	Response to oxidative stress	0. 0166	
	Response to ethylene	0. 0228	
	Cellular oxidant detoxification	0. 0235	
	Inositol trisphosphate metabolic process	0. 0035	
	Caffeoyl-CoA O-methyltransferase activity	0. 0009	
	Symporter activity	0. 0013	
	Protein serine/threonine phosphatase activity	0. 0014	
	Inositol tetrakisphosphate 1-kinase activity	0. 0032	
	Peroxidase activity	0. 0098	
	Protein kinase binding	0. 0261	
	calmodulin binding	0. 0266	
	Response to hydrogen peroxide	0. 0100	
Antiquewhite1	Regulation of mRNA stability	1.34--05	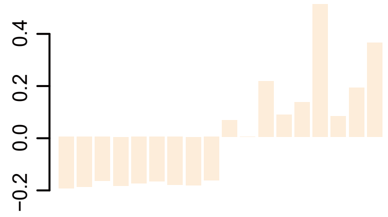
	Terpene synthase activity	2.64E-05	
	Geranyl diphosphate metabolic process	3.15E-05	
	Glycogen catabolic process	0.0007	
	Response to water deprivation	0.0014	
	Terpenoid biosynthetic process	0.0017	
	Intracellular signal transduction	0.0029	
	Glycogen phosphorylase activity	0.0013	
Coral1	Drug transmembrane transport	2. 09E- 11	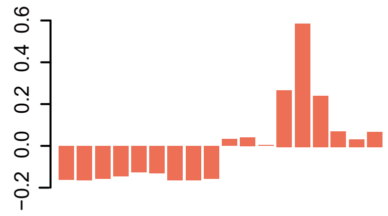
	Oxidation-reduction process	2. 06E- 09	
	Unsaturated fatty acid biosynthetic process	9. 91E- 09	
	Lipid metabolic process	1. 01E- 05	
	S-adenosylmethioninamine biosynthetic process	1. 94E- 05	
	Spermine biosynthetic process	1. 94E- 05	
	Spermidine biosynthetic process	0. 0002	
	Anion transmembrane transport	9. 70E- 05	
	Cellular ion homeostasis	0. 0001	
	Brassinosteroid biosynthetic process	0. 0001	
	Brassinosteroid homeostasis	0. 0001	
	Organic anion transport	0. 0011	
	Protein targeting to chloroplast	0. 0011	
	Stomatal closure	0. 0160	
	Photosystem II repair	0. 0222	
	Signal transduction	0. 0375	
	Response to carbon dioxide	0. 0034	
	Carbon utilization	0. 0028	
	Ion transport	0. 0144	
Orangered4	trehalose biosynthetic process	0.0002	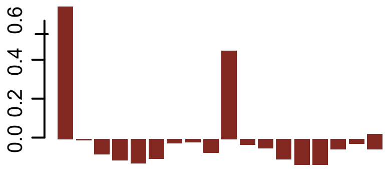
	lactate biosynthetic process	0.0002	
	protein metabolic process	0.0003	
	mRNA splicing, via spliceosome	0.0004	
Indianred4	Cellulose catabolic process	0.0017	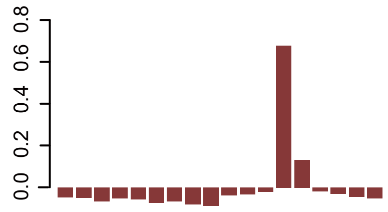
	Phosphorylation	0.0223	
	Lipid metabolic process	0.0420	
Powderblue	Cinnamic acid biosynthetic process	2.40E-16	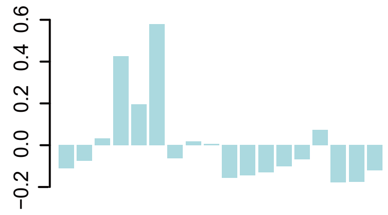
	L-phenylalanine catabolic process	1.19E-15	
	Methylation	5.33E-13	
	Methionine biosynthetic process	1.87E-08	
	One-carbon metabolic process	8.49E-05	
	S-adenosylmethionine cycle	0.0004	
	Iron ion transport	0.0007	
	Lignin biosynthetic process	0.0010	
	Unsaturated fatty acid biosynthetic process	0.0031	
	Cellular iron ion homeostasis	0.0032	
	Oxidation-reduction process	0.0171	
	Flavonoid biosynthetic process	0.0186	
Indianred2	rRNA processing	2.22E-07	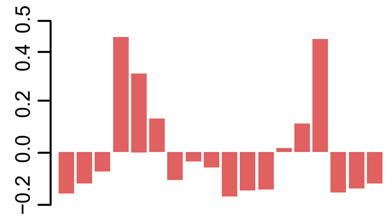
	tRNA export from nucleus	9.84E-06	
	Protein folding	5.44E-05	
	Glycolytic process	0.0004	
	Ribosome biogenesis	0.0006	
	Response to water	0.0009	
	Cellulose biosynthetic process	0.0039	
	Plant-type cell wall biogenesis	0.0042	
Purple	Lipid transport	7.43E-13	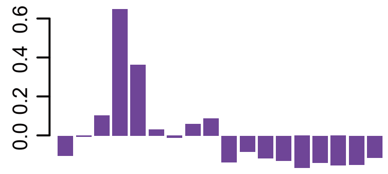
	Hydrogen peroxide catabolic process	3.06E-08	
	Cellular oxidant detoxification	2.73E-07	
	Phenylpropanoid biosynthetic process	9.86E-06	
	Cutin biosynthetic process	0.0002	
	Plant-type cell wall organization	0.0002	
	T-adenosylmethionine biosynthetic process	0.0003	
	Cellular glucose homeostasis	0.0093	
	Heat acclimation	0.0106	
	Response to osmotic stress	0.0448	
	Peroxidase activity	1.19E-08	
	Fatty-acyl-CoA reductase	0.0001	
Darkgreen	transport	1.12E-06	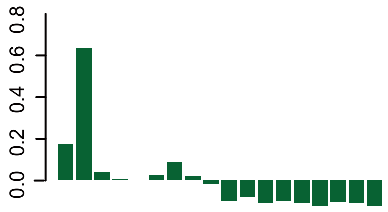
	cellular ion homeostasis	0.0002	
	transmembrane transport	0.0006	
	phosphatidylinositol dephosphorylation	0.0002	
	transporter activity	3.46E-10	
	integral component of membrane	4.03E-07	
