# Supplementary material for: Transcriptome dynamics in Artemisia annua provides new insights into cold adaptation and de-adaptation
Source: Front Plant Sci. 2024 Aug 29;15:1412416. doi: 10.3389/fpls.2024.1412416 (PMC11390472; doi:10.3389/fpls.2024.1412416)
Supplement: Supplementary file 1 [file DataSheet1.zip › Supplementary Table/Supplementary Table 7.pdf]

Supplementary Table S7. KEGG Enrichment of DEGs at RD2 in leaves

Kegg_pathway	KO_ID	Cluter_frequency	Genome_frequency	P-value
		Up DEGs		
Flavonoid biosynthesis	ko00941	10 out of 212 4.7%	145 out of 13590 1.1%	9.12E-05
Circadian rhythm - plant	ko04712	7 out of 212 3.3%	87 out of 13590 0.6%	0.000413797
Phenylpropanoid biosynthesis	ko00940	22 out of 212 10.3%	687 out of 13590 5.0%	0.001094758
Stilbenoid, diarylheptanoid and gingerol biosynthesis	ko00945	7 out of 212 3.3%	110 out of 13590 0.8%	0.001667941
Base excision repair	ko03410	6 out of 212 2.8%	95 out of 13590 0.6%	0.003687764
Phenylalanine metabolism	ko00360	7 out of 212 3.3%	135 out of 13590 0.9%	0.005240858
beta-Alanine metabolism	ko00410	6 out of 212 2.8%	106 out of 13590 0.7%	0.006289376
		Down DEGs		
Circadian rhythm - plant	ko04712	11 out of 185 5.9%	87 out of 13590 0.6%	2.52E-08
Plant-pathogen interaction	ko04626	15 out of 185 8.1%	407 out of 13590 2.9%	0.000455186
Biosynthesis of unsaturated fatty acids	ko01040	7 out of 185 3.7%	125 out of 13590 0.9%	0.001599014
Linoleic acid metabolism	ko00591	4 out of 185 2.1%	41 out of 13590 0.3%	0.00227183
alpha-Linolenic acid metabolism	ko00592	7 out of 185 3.7%	140 out of 13590 1.0%	0.00303898
Thiamine metabolism	ko00730	3 out of 185 1.6%	27 out of 13590 0.1%	0.00570857
Butanoate metabolism	ko00650	4 out of 185 2.1	55 out of 13590 0.4%	0.006603085
Fatty acid metabolism	ko01212	10 out of 185 5.4%	302 out of 13590 2.2%	0.008425728
