# Supplementary material for: Transcriptome dynamics in Artemisia annua provides new insights into cold adaptation and de-adaptation
Source: Front Plant Sci. 2024 Aug 29;15:1412416. doi: 10.3389/fpls.2024.1412416 (PMC11390472; doi:10.3389/fpls.2024.1412416)
Supplement: Supplementary file 1 [file DataSheet1.zip › Supplementary Table/Supplementary Table 8.pdf]

Supplementary Table 8. GO Enrichment of DEGs at RD2 in leaves

GO_ID	GO_term	Items	Background Items	FDR
	Up DEGs			
GO:0000786	nucleosome	18	171	~0
GO:0006334	nucleosome assembly	16	143	~0
GO:0046982	protein heterodimerization activity	18	228	2.63E-11
GO:0016168	chlorophyll binding	7	77	9.55E-06
GO:0003677	DNA binding	25	1614	2.03E-05
GO:0009522	photosystem I	7	82	2.25E-05
GO:0009535	chloroplast thylakoid membrane	10	271	0.000172181
GO:0018298	protein-chromophore linkage	5	81	0.004515958
GO:0005634	nucleus	29	2554	0.006828018
GO:0009059	macromolecule biosynthetic process	2	3	0.007160068
GO:0009768	photosynthesis, light harvesting in photosystem I	3	32	0.049958035
	Down DEGs			
GO:0009916	alternative oxidase activity	5	10	5.91E-05
GO:0004402	histone acetyltransferase activity	5	19	0.00242776
GO:0016717	oxidoreductase activity, acting on paired donors	8	68	0.003033673
GO:0006355	regulation of transcription, DNA-templated	41	1268	0.004857549
GO:0052837	thiazole biosynthetic process	3	4	0.00488651
GO:0043565	sequence-specific DNA binding	18	376	0.008953351
GO:0003712	transcription cofactor activity	5	26	0.012541706
GO:0070469	respiratory chain	5	40	0.029475654
GO:0003700	transcription factor activity, sequence-specific DNA binding	22	571	0.032459729
GO:0009228	thiamine biosynthetic process	3	7	0.04125242
