# Supplementary material for: Transcriptome dynamics in Artemisia annua provides new insights into cold adaptation and de-adaptation
Source: Front Plant Sci. 2024 Aug 29;15:1412416. doi: 10.3389/fpls.2024.1412416 (PMC11390472; doi:10.3389/fpls.2024.1412416)
Supplement: Supplementary file 1 [file DataSheet1.zip › Supplementary Table/Supplementary Table 9.pdf]

Supplementary Table 9. Primers for RT-qPCR

Gene	Forward primer	Reverse primer
PAL	CATAGAAGCCATCCCGGACC	GCTGGTGTAACTACCGGCTT
LOX	ATGGTGGACCGAGATCAGGA	GTGGTGTTTTGAGAACGGGC
DREB	GGTTGCCTGGTGTTATTGGC	CCATGCCCGACTGTATGAGG
COR	GTCCTTCAATGGTTCGCGTG	GGAAATGCCGTTGCTCTTGG
ELIP	TTAGCATTCAGTGGGCCAGC	CCCAATGCTGACACAAACCC
AMD1	AGGGAGAGAGCATCCGTCTT	TCAAAACATGCCAGCACACG
CHS	CCATTCTAGCGATCGGCACT	TTGCGGCTTCTTTTCCAAGC
Aquaporin	CGTTCCAATTTTGGCACCGT	TTGCCACTACACGACTCACC
FAD	TGTGGCATGGCCGATTTACT	GAGTATCTGGAGGCGCTCAC
HY5	GATGGCAGCAACAAAGAGGC	CGCGTTTTTCGGGACCATTT
COP1	GTACCAACTCCAGCTGACCC	GCAGGAGCTTTTCCATCCCT
ACTIN	CCAGGCTGTTCAGTCTCTGTATGC	CGAAGGATGGCGTGTGGAAGG
