# Supplementary figures and images for: Transcriptome dynamics in Artemisia annua provides new insights into cold adaptation and de-adaptation
Source: Front Plant Sci. 2024 Aug 29;15:1412416. doi: 10.3389/fpls.2024.1412416 (PMC11390472; doi:10.3389/fpls.2024.1412416)

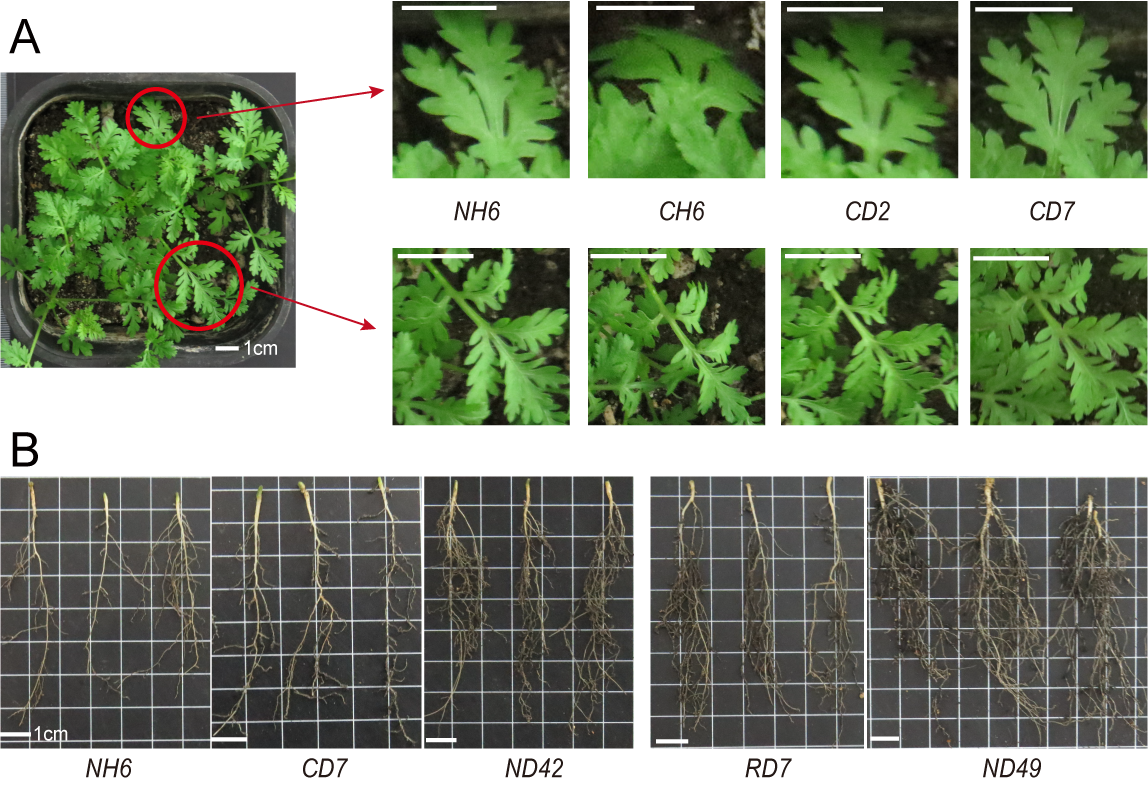

Supplement: Supplementary Figure 1 — (A) Morphological traits of leaves. (B) Morphological traits of roots. [file Image1.tif]

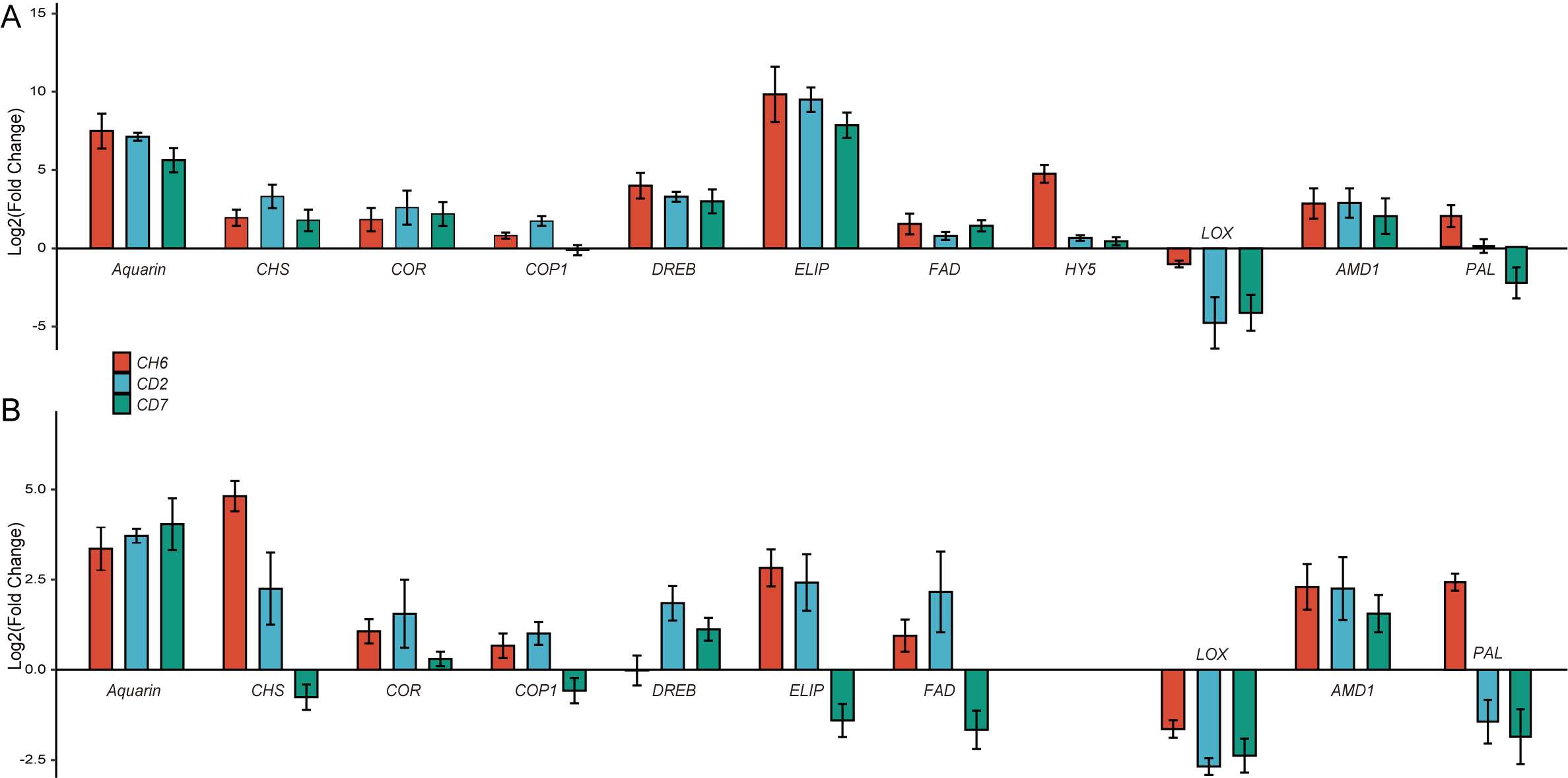

Supplement: Supplementary Figure 2 — Validation of 10 gene expression levels under cold stress using quantitative real-time PCR. (A, B) Bars in leaves and roots represent the log2 values of fold changes of the cold stress samples compared to the NH6 (i.e., take the gene expression level of NH6 as 1; three biological replicates). Values are expressed as mean ± SE (n = 3). [file Image2.tif]

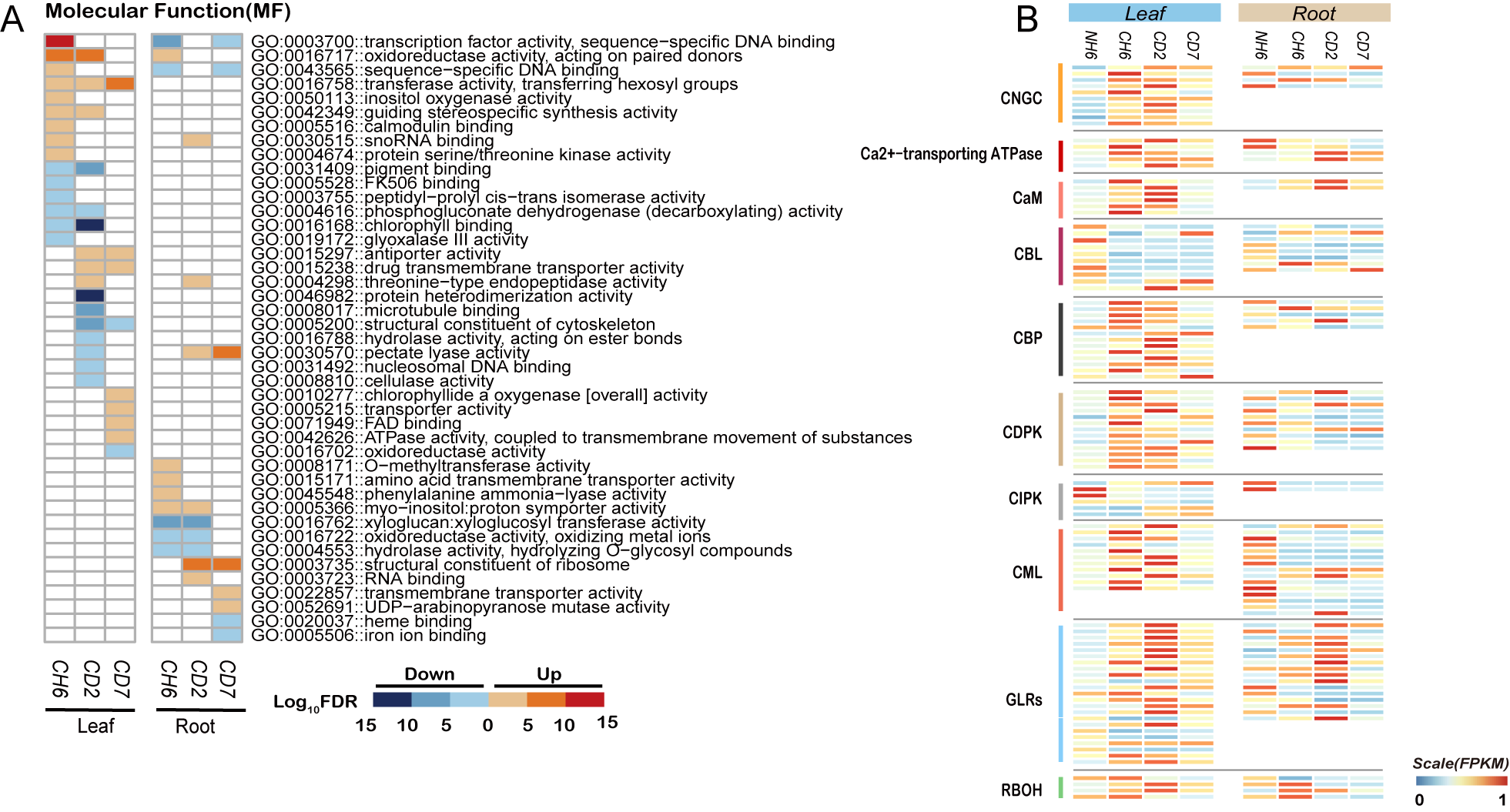

Supplement: Supplementary Figure 3 — (A) Heatmaps illustrating non-redundant Gene Ontology term enrichment (MF; Molecular Function; FDR < 0.05). The color scale corresponds to the -log10 transformation of the FDR for the enrichment according to Fisher’s exact test. The regulatory trend of the transcripts in each bin (up or down) is indicated. (B) Heatmaps illustrating DEGs related to calcium channel and kinase proteins in leaves and roots under cold treatment. [file Image3.tif]

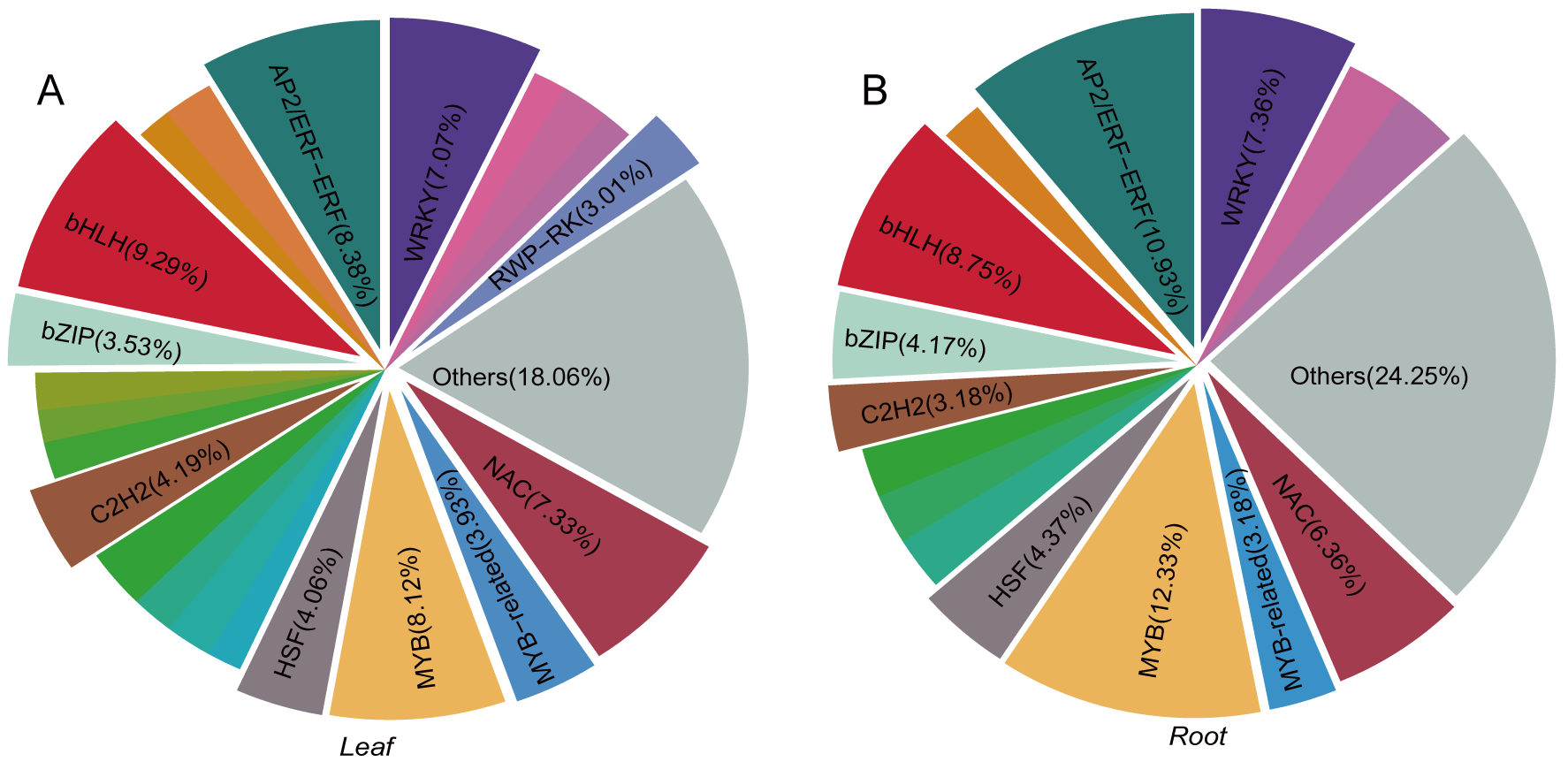

Supplement: Supplementary Figure 4 — Analysis of transcription factors (TFs) in differentially expressed genes (DEGs). (A) Proportion of major differential transcription factors in leaves. (B) Proportion of major differential transcription factors in roots. [file Image4.tif]

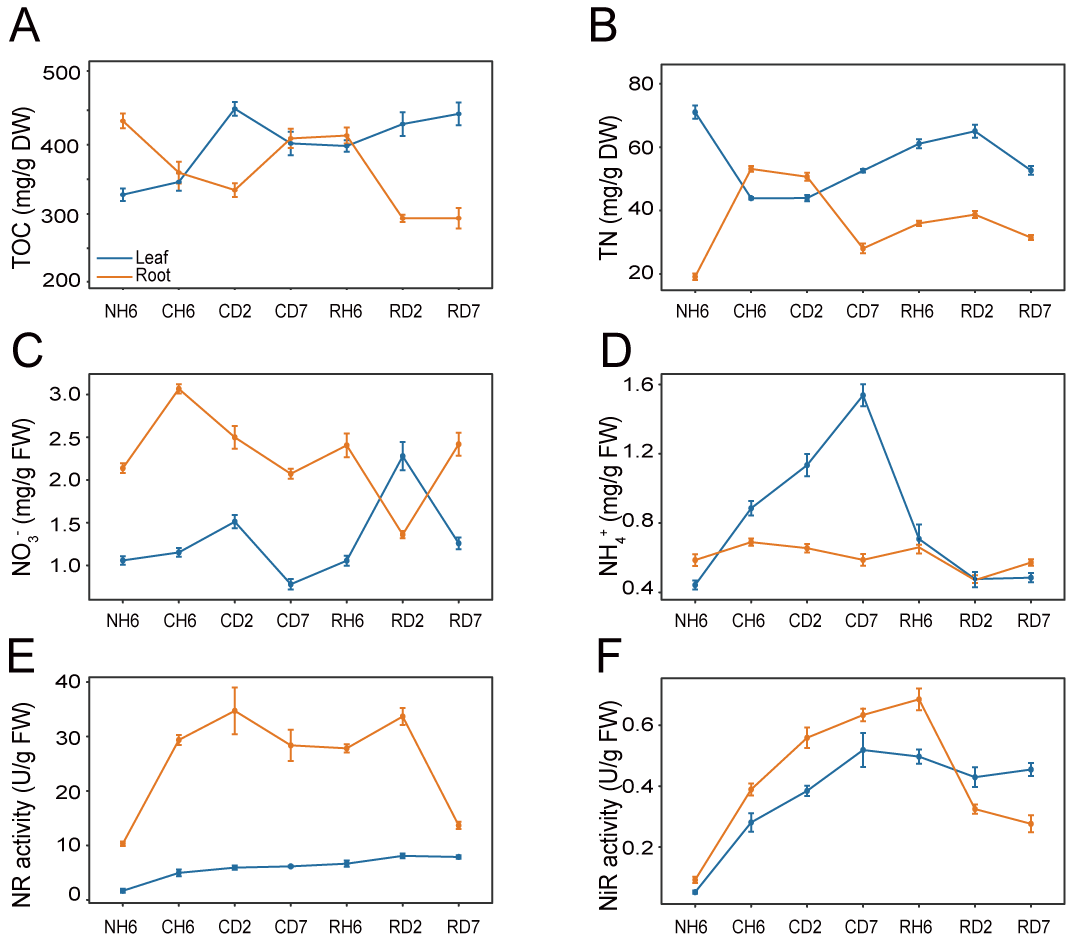

Supplement: Supplementary Figure 5 — (A, B) Dynamics of total organic carbon (TOC; A) and total nitrogen (TN; B) of leaves and roots throughout the experiment. (C, D) Nitrate nitrogen (NO3-; C) and ammonium nitrogen content (NH4+; D). (E, F) Nitrate reductase (NR; E) and nitrite reductase (NiR; F) activity. The abbreviations of DW and FW represent dry weight and fresh weight, respectively. [file Image5.tif]

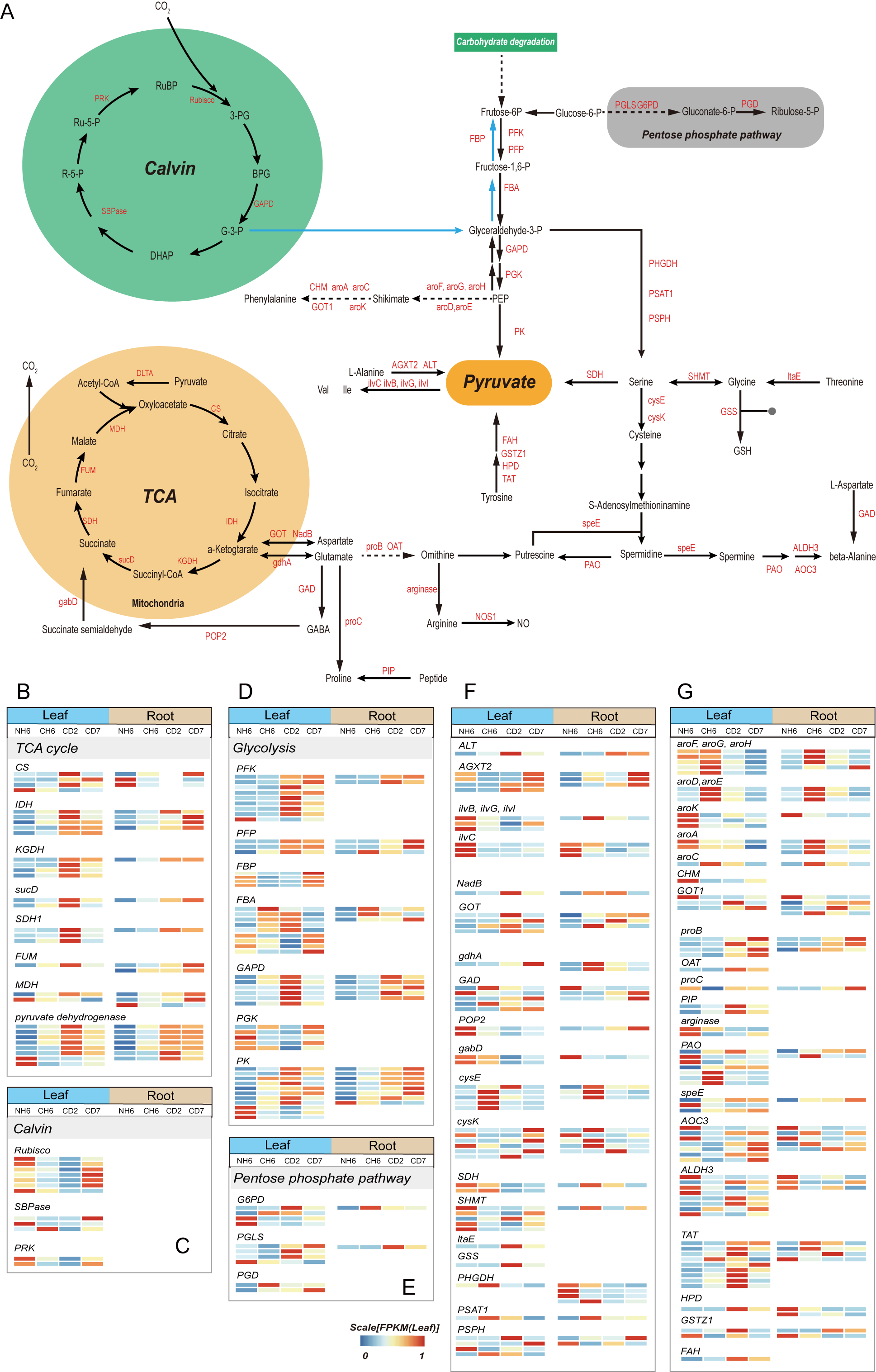

Supplement: Supplementary Figure 6 — Co-expression analysis of carbohydrate metabolism and amino acid metabolism in leaves and roots under cold treatment. [file Image6.tif]

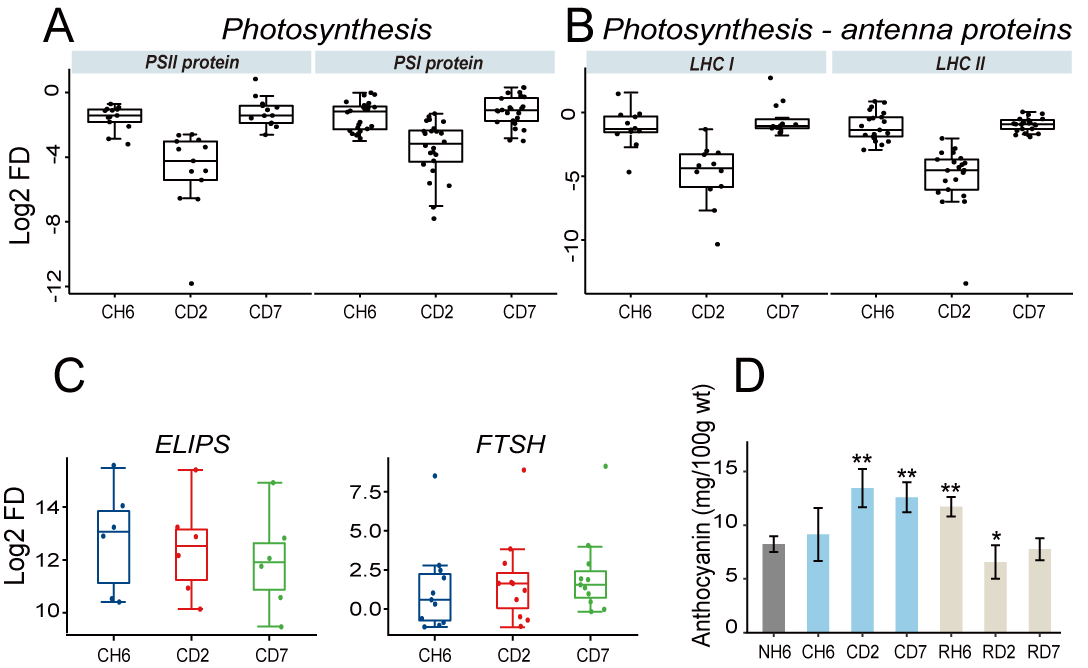

Supplement: Supplementary Figure 7 — (A, B) Comparison of the expression of PSII and PSI genes, LHCI and LHCII genes annotated by the KEGG database under cold stress. (C) Boxes showing the expression of ELIPs and FTSH compared with NH6 under cold stress. Boxes represent the log2 values of fold changes of the cold stress samples compared to the NH6. (D) Anthocyanin content in leaves. Asterisks indicate significant differences between treatment groups and the control group (NH6). Significance levels are: *P < 0.05; **P < 0.01. [file Image7.tif]

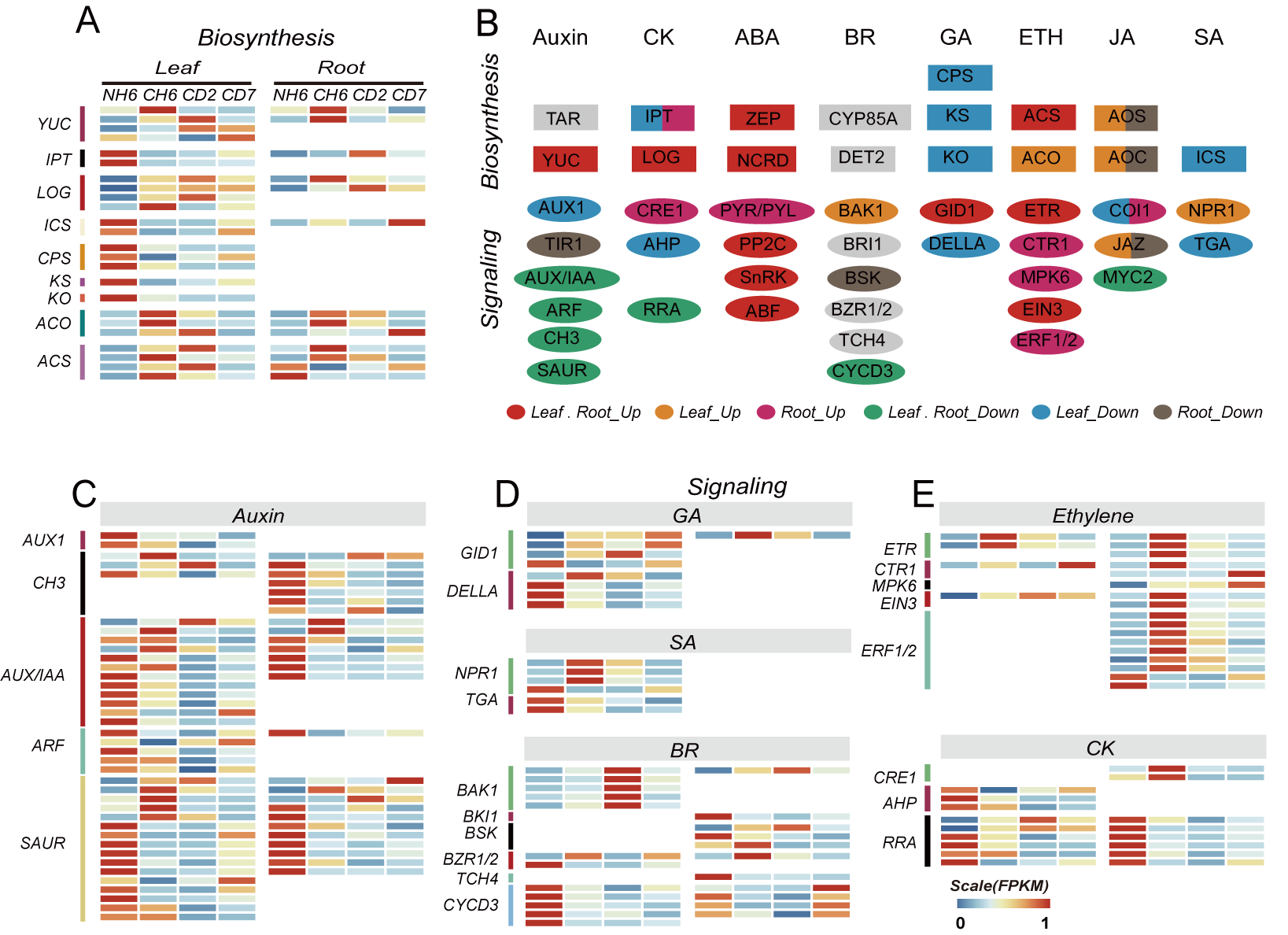

Supplement: Supplementary Figure 8 — (A) Changes in the expression of DEGs involved in Auxin, GA, SA, Ethylene, BR and CK biosynthesis in leaves and roots under cold treatment. (B) The picture representing changes in various hormone synthesis and signaling pathways. (C–E) Changes in the expression of DEGs in Auxin, GA, SA, Ethylene, BR and CK signal pathways in leaves and roots under cold treatment. CK, Cytokinin; ABA, Abscisic acid; BR, Brassinosteroids; GA, Gibberellic acid; ETH, Ethylene; JA, jasmonic acid; SA, Salicylic acid. [file Image8.tif]
